# Dust storms: Hidden drivers of extreme rainfall and global precipitation shifts

**DOI:** 10.1126/sciadv.adw6864

**Published:** 2026-04-29

**Authors:** Yuzhi Liu, Weiqi Tang, Tianbin Shao, Run Luo, Ziyuan Tan, Dan Li, Jianping Huang

**Affiliations:** ^1^ Key Laboratory for Semi-Arid Climate Change of the Ministry of Education, Lanzhou University, Lanzhou 730000, China.; ^2^ Collaborative Innovation Center for Western Ecological Safety, Lanzhou University, Lanzhou 730000, China.; ^3^ Weather Modification Center (WMC), China Meteorological Administration (CMA), Beijing 100000, China.; ^4^ Liupanshan Atmospheric Science Field Observation and Research Station in Ningxia Hui Autonomous Region, Yinchuan 750002, China.

## Abstract

Dust storms, while often seen as harmful, can play an unexpected role in enhancing rainfall. Global observations show that 7-day accumulated precipitation after dust storms exceeds dust-free conditions by up to 9.6 millimeters. Numerical simulations further confirm that dust particles act as ice nuclei, thereby promoting cloud formation and increasing rainfall through the ice crystal effect. Moreover, in regions with rising anthropogenic aerosols, dusts determine precipitation patterns. While elevated levels of anthropogenic aerosols alone tend to boost weak rainfall, the presence of dust aerosols reduces light precipitation and enhances heavier precipitation. Collectively, these findings reveal a dual role of dust storms in shaping global precipitation patterns while adversely affecting the human living environment. This research establishes a mechanistic framework for understanding how dust affects extreme precipitation at the global scale, advancing predictive capabilities for heavy precipitation.

## Teaser

Dust storms boost rain via ice nucleation effects, reshaping precipitation patterns globally.

## INTRODUCTION

Dust storms represent a distinctive type of natural disaster, characterized by the transport of vast quantities of dust particles from source regions, exerting far-reaching effects on broader regions. These dust events can cause extensive disruptions and pose substantial risks to both socioeconomic systems and human health (*1*, *2*). For example, dusts originating in Northwest China can travel as far as the North Pacific, Japan, and Korea. They may even cross the Pacific Ocean, reaching the western coast of North America (*3*–*5*); dusts transported from North Africa and the Middle East can traverse vast distances, ultimately influencing atmospheric conditions as far as East Asia (*6*, *7*). While previous research has predominantly examined the characteristics and trends of dust storms in specific areas, such as northern China (*8*–*10*) and North Africa (*11*), studies on the global variability of dust storms remain scarce.

Dust particles affect temperature and atmospheric stability by scattering and absorbing solar radiation (*12*–*18*). Besides, as cloud condensation nuclei (CCN) or ice nuclei (IN), they influence cloud properties and precipitation (*19*–*30*). In summary, dust aerosols exert both direct and indirect effects on Earth-atmosphere radiation balance and rainfall processes. Their absorptive properties enable them to trap shortwave solar radiation within dust-laden atmospheric layers, thereby exerting dual thermodynamic effects, cooling the surface while simultaneously heating the atmosphere (*13*, *31*). Simultaneously, dust aerosols serve as efficient ice-nucleating particles, actively modulating precipitation processes and radiation budget indirectly through their ability to initiate ice formation in clouds (*23*, *25*, *27*, *29*). Despite these impacts, the intricate interactions between dust and cloud microphysics make the effects of dust on precipitation poorly understood (*32*–*35*).

Overall, previous studies have examined the effects of dust on precipitation in specific regions, such as Northwest China (*36*), the Tibetan Plateau (*25*–*29*), the Tarim Basin (*37*), and the continental Northern Hemisphere (*38*). However, understanding of global dust event distribution and its influence on precipitation remains limited. In this study, we explore the global variability of dust events and their relationship with precipitation by integrating site observations, satellite data, reanalysis products, and numerical model simulations.

## RESULTS

### Interdecadal variability of global dust events

**Fig. 1.**
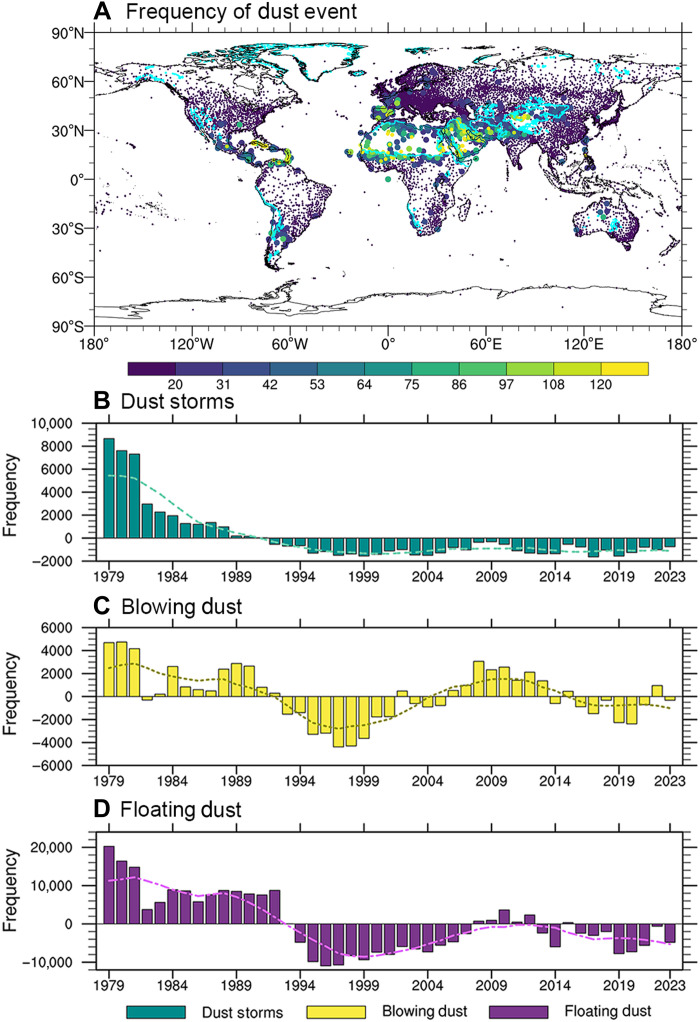
Spatial and temporal patterns of global dust events. (**A**) Global average frequency of dust events (including dust storms, blowing dust, and floating dust) from 1979 to 2023. The cyan lines in (A) delineate the boundaries between dust source regions and transport regions. The largest markers indicate stations where dust storms are the dominant type of dust events, medium-sized markers represent blowing dust, and the smallest markers denote floating dust. (**B**), (**C**), and (**D**) present the global frequency anomaly time series for dust storms, blowing dust, and floating dust, respectively, over the same period. The curves in [(B), (C), and (D)] are smoothed using a nine-point moving average.

Figure 1 illustrates the observed frequency and primary types of “dust event” (including dust storms, blowing dust, and floating dust; definitions are detailed in Materials and Methods) across the global dust source regions (enclosed by the cyan line) and transport regions (outside the cyan line) (Details regarding the delineation method and spatial distributions of source and transport regions are provided in text S1 and fig. S1). As shown in Fig. 1A, dust events predominantly occur within the dust source regions. In regions including North Africa, the Arabian Peninsula, the Iranian Plateau, and East Asia, the frequency of dust events surpasses 120 days annually. Moreover, dust source regions are mainly characterized by dust storms and blowing dust. Specifically, dust storms dominate in the Gobi Desert, the Thar Desert, the Taklimakan Desert, and the deserts of the Arabian Peninsula. In contrast, the western Sahara Desert experiences primarily blowing dust, while the eastern Sahara is dominated by dust storms.

The time series of global dust storms, blowing dust, and floating dust frequencies from 1979 to 2023 are shown in Fig. 1 (B to D). Globally, dust events exhibit an interdecadal oscillation with a cycle of approximately 14 years, with more pronounced cyclic patterns for floating and blowing dust in source regions (fig. S2). Over the first two decades of the study period, the frequency of dust storms declined notably (Fig. 1B). However, unlike the global trend, dust storms in source regions increased after 1998 and then declined again after 2011 (fig. S2A). In contrast, in dust transport regions, the frequency of dust storms, blowing dust, and floating dust consistently decreased between 1979 and 1998 (fig. S3).

### Observed enhancement of precipitation after dust events

**Fig. 2.**
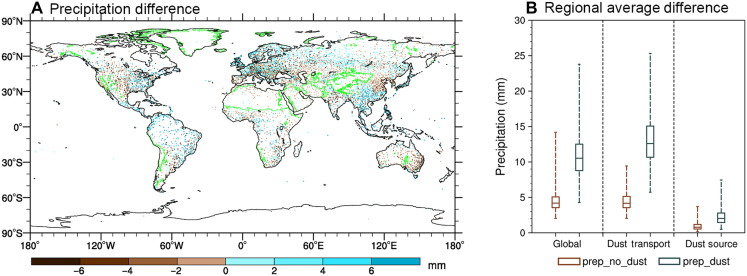
Impact of dust on 7-day accumulated precipitation. (**A**) Global differences in 7-day accumulated precipitation (in millimeters) between scenarios with and without dust events during 1979–2023. The green lines in (A) delineate the boundaries between dust source regions and transport regions (same as the cyan lines in Fig. 1A). (**B**) Regional averages of 7-day accumulated precipitation differences (in millimeters) under the same conditions, analyzed globally, in dust transport regions, and in dust source regions. Green boxes represent precipitation following dust events (prep_dust), while brown boxes represent precipitation without dust events (prep_no_dust). The boxes illustrate the interquartile range (25th to 75th percentiles), with the median marked by a line inside each box. The whiskers denote the minimum and maximum values.

To estimate the impact of dust aerosols on precipitation, we statistically calculate the difference in accumulated precipitation over the 7 days (hereafter termed 7-day accumulated precipitation) after dust events and dust-free days on a global scale (detailed methods are described in Materials and Methods and text S2). Figure 2A shows the 1979–2023 mean difference in 7-day accumulated precipitation between dust events and dust-free conditions, approximately reflecting dust-precipitation interactions. Positive anomalies (cyan) indicate enhanced precipitation after dust events, while negative anomalies (brown) indicate suppressed precipitation. As shown in Fig. 2A, the predominance of cyan regions demonstrates systematic precipitation increases following dust events, although with some regional heterogeneity. Within dust source regions, the 7-day accumulated precipitation after dust events is higher in regions including the southern Sahara Desert and the Taklimakan Desert while lower in the Gobi Desert, Thar Desert, Arabian Desert, and the deserts of the Iranian Plateau. In dust transport regions, the 7-day accumulated precipitation is higher in some areas with infrequent dust events, such as South America and South Asia (see Figs. 1A and 2A). However, despite regional variations, the overall enhancement of 7-day accumulated precipitation following dust events compared to dust-free conditions remains statistically significant.

On average, the 7-day accumulated precipitation following dust events is higher than that without dust events, as illustrated in Fig. 2B. While the spatial distribution of 7-day accumulated precipitation after dust events and dust-free days remains similar, higher precipitation is observed following dust events (fig. S4). At the global scale, the 7-day accumulated precipitation following dust events reaches up to 23.79 mm, which is 9.6 mm more than when no dust event occurs (calculation methods are provided in text S3). In dust source regions, although average precipitation is lower, the 7-day accumulated precipitation following dust events shows an overall higher value than in dust-free conditions. In dust transport regions, precipitation following dust events is notably higher, with a maximum of 25.32 mm observed. Overall, the 7-day accumulated precipitation following dust events is remarkably higher than during dust-free periods, a phenomenon primarily attributed to the role of dust aerosols in affecting precipitation. From a physical perspective, dust aerosols act as efficient ice-nucleating particles, promoting the microphysical processes in ice-phase and mixed-phase clouds, thereby affecting precipitation (*25*, *27*, *37*).

Although 7-day accumulated precipitation after dust events is notably enhanced compared to dust-free periods, it is important to note that aerosols represent only one factor in cloud and precipitation formations, with other conditions, such as water vapor availability and dynamic conditions, also playing critical roles. To disentangle the meteorological confounding effects, days with the highest number of global sites simultaneously experiencing dust events (hereafter referred to as “dust-active days”) and those without dust events (hereafter termed “dust-inactive days”) are further selected for composite analysis (statistical details are listed in table S1). As shown in fig. S5, 7-day accumulated precipitation systematically increases after dust events, with green markers consistently exceeding brown markers. This observed precipitation difference suggests a potential linkage between atmospheric dust loading and subsequent precipitation processes. To clarify the impact of meteorological conditions on the differences in precipitation between dust-active days and dust-inactive days, a composite analysis of the meteorological fields under both scenarios is conducted (the method is described in text S4).

**Fig. 3.**
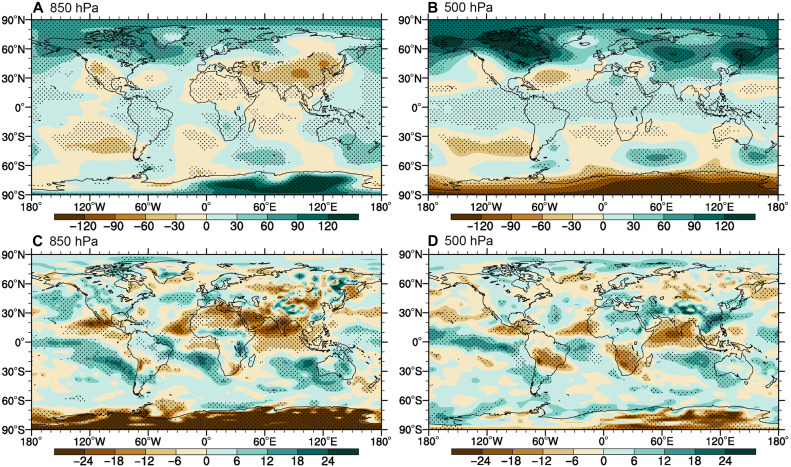
Atmospheric anomalies associated with dust activity. (**A** and **B**) Composite differences in 850- and 500-hPa geopotential height (contours) between dust-active and dust-inactive periods. (**C** and **D**) Corresponding relative humidity anomalies (in percent; color shading). Black dots indicate anomalies significant at the 95% confidence level in both pointwise and field significance tests.

Figure 3 presents the composite field anomalies of geopotential height, wind, and relative humidity between dust-active and dust-inactive conditions, with significance assessed through both pointwise and field significance tests (text S5). Negative geopotential height anomalies at 850 hPa over major dust source regions (e.g., the eastern Sahara Desert and Taklimakan Desert) indicate anomalous cyclonic activity during dust-active days (Fig. 3A and fig. S6A), suggesting enhanced upward motion. However, low-level moisture exhibits negative anomalies (850 hPa; Fig. 3C; similar at 700 hPa, figure omitted), implying drier lower-atmospheric conditions despite stronger dynamical lifting. This moisture deficit likely limits the potential for enhanced precipitation, even with favorable vertical motion. At 500 hPa, dust-active days feature easterly wind anomalies, cold advection (Fig. 3B and fig. S6B), and elevated relative humidity (Fig. 3D) over dust source regions. Dust transport regions show similar but spatially variable patterns of the anomalies. Collectively, the composite analysis of meteorological fields demonstrates that, from a purely dynamical perspective, dust-active days do not exhibit more favorable meteorological conditions for precipitation formation compared to dust-inactive days.

### Simulated enhancement of precipitation by dust-cloud interactions

To validate the statistical results in Fig. 2, several numerical simulations are performed to examine the effects of dust aerosols on the precipitation process in both the dust source and transport regions (refer to Materials and Methods for details on model description). The Northwestern region of China, home to the Taklimakan Desert, is chosen as a representative dust source region, while North China is selected as the dust transport region (fig. S7).

**Fig. 4.**
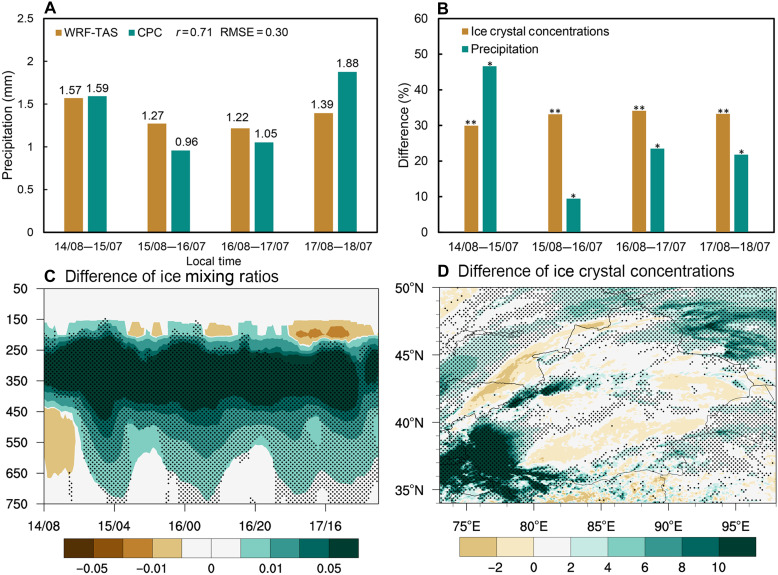
Ensemble-mean effects of dust doubling on cloud microphysics and precipitation from perturbed initial conditions during the 14 to 17 April 2020 dust storm (Dust_1). (**A**) illustrates model-simulated and observed regional average daily accumulated precipitation (unit: in millimeters). (**B**) presents the percentage differences in daily average ice crystal number concentrations and precipitation in the dust source region between the doubling dust experiment and the control experiment. (**C**) indicates the impacts of dust aerosols on ice mixing ratios (unit: in milligrams per kilogram) during period from 14 to 17 April 2020. (**D**) shows the effects of dust serving as IN on ice crystal number concentrations (units: 10^4^ kg^−1^) during period from 14 to 17 April 2020. In (B), single asterisk (*) above bars denote significance at the 90% confidence level in paired tests, while double asterisks (**) indicate 95% confidence. In (C) and (D), black dots represent results passing paired *t* tests at the 95% significance level. RMSE, root mean square error.

Figure 4 presents the Weather Research and Forecasting (WRF) model simulated impact of dust on cloud microphysics and precipitation during 14 to 17 April 2020 (Dust_1) in the source region (model configuration is described in Materials and Methods and table S2). To quantify the dust-induced impacts on cloud and precipitation processes, paired ensemble simulations are conducted for the Dust_1 event under 1× dust (control experiment) and 2× dust (sensitive experiment) concentration conditions (detailed in text S6 and table S3). Each 10-member ensemble used identical initial condition perturbations across corresponding member pairs, enabling direct comparison between the two aerosol scenarios. Consequently, the differences between the control and sensitive ensembles demonstrate the aerosol effects on cloud microphysics and precipitation. The statistical significance of these differences is confirmed through paired *t* tests applied to matched ensemble member differences (methods are detailed in text S7).

The model’s daily precipitation output is first validated against observational data from the Climate Prediction Center (CPC) (Fig. 4A). The results show that the regional daily mean precipitation ranges between 1.22 to 1.57 mm (WRF simulations) and 0.96 to 1.88 mm (CPC observations), demonstrating that the model accurately captures precipitation feature during the period. More comprehensive model evaluation results are presented in figs. S8 and S9. Furthermore, from a temporal perspective, as shown in Fig. 4B, the ice crystal concentration demonstrates a continuous rise in this typical dust event, peaking at over 29%. Even after 17th April, the ice crystal concentration remains above the background level, although the rate of increase diminishes. On average, precipitation during dust events rises by 25.3% compared to conditions with background dust concentrations, demonstrating a statistically significant positive effect on precipitation enhancement.

From the perspective of the spatial distribution of ice particles in clouds, simulations for the dust source region reveal that higher dust concentrations lead to a notable enhancement in the concentration of ice crystals within clouds (Fig. 4, C and D) due to the dusts’ indirect effect. Figure 4C quantifies dust-induced modifications to the mass mixing ratio of ice in clouds, indicating a robust enhancement throughout the atmospheric column, particularly pronounced between 450 and 250 hPa. This positive perturbation exhibits temporal consistency except for transient, spatially confined reductions observed after 17 April. As shown in Fig. 4D, the concentration of ice crystals on the western side of the Taklimakan Desert can reach up to 10 × 10^4^ kg^−1^ as dust concentration rises. While the effect of dusts on ice crystal concentration differs between the southern and northern sides of the desert, overall, increased dusts remarkably enhance the number of ice particles in clouds.

In the dust transport region of North China, characterized by intense human activity and widespread anthropogenic aerosols, the influence of transported dust aerosols on cloud precipitation exhibits notable variability. To investigate this, a representative dust transport event occurring from 22 to 27 April 2014 (Dust_2) is simulated using the same modeling framework applied to the Dust_1 event. Random perturbations in the initial fields are included too, following the approach described in Materials and Methods and tables S2 and S3. During this period, a severe Mongolian dust event transported substantial dust aerosols to North China, peaking on 24 to 25 April. In the simulations, anthropogenic aerosols, given their substantial background levels, are parameterized as hydrophilic particles at fixed concentrations across experiments, ensuring that differences between double-dust and control simulations resulted solely from dust perturbations.

**Fig. 5.**
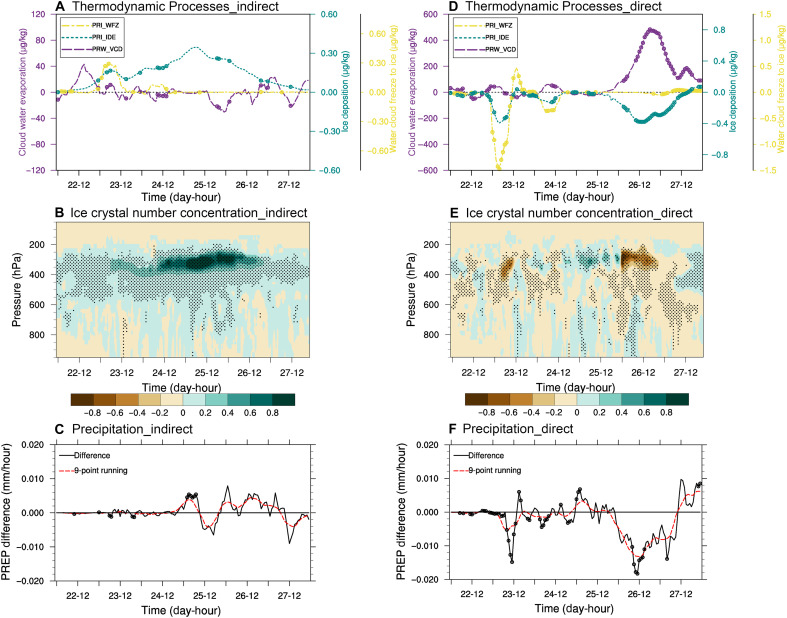
Ensemble-mean effects of dust doubling on cloud microphysics and precipitation from initial-condition perturbation experiments during the dust event from 22 to 27 April 2014 (Dust_2). Indirect effects: (**A**) changes in cloud droplet evaporation (purple), ice deposition (cyan), and freezing (yellow); (**B**) change in ice crystal number concentrations; (**C**) change in precipitation. Direct effects: (**D** to **F**) corresponding changes as in (A) to (C). In (A), (C), (D), and (F), open circles (○) indicate statistical significance at the 90% confidence level in paired tests, while solid dots (•) in (B) and (E) denote significance at the 95% confidence level. In (B) and (E), black dots represent results passing paired *t* tests at the 95% significance level. PREP, precipitation.

Dust aerosols exert both direct effects by altering radiative balance through absorption and scattering processes and indirect effects by serving as IN that modify cloud microphysical processes. Specifically, the indirect effects of dust aerosols enhance ice deposition while simultaneously increasing the evaporation of cloud droplets (Fig. 5A). This process facilitates the transformation of larger cloud droplets into smaller ones, promoting coalescence growth and the subsequent formation of raindrops exceeding 40 μm in diameter. Furthermore, sustained cloud droplet evaporation maintains elevated water vapor content and relative humidity within the clouds, thereby inhibiting the evaporation of large raindrops. In contrast, the direct radiative effect of dust—mediated through its limited solar absorption capacity—induces lower atmospheric warming (fig. S10). This warming suppresses ice nucleation and deposition processes while intensifying the evaporation of cloud droplets (Fig. 5D). When both the direct and indirect effects of dust are taken into account, the increase in ice deposition caused by dust is also the highest during the peak rainfall period (fig. S11A).

Furthermore, as illustrated in Fig. 5B, the dust indirect effect enhances the concentration of ice crystals in clouds, with the most pronounced increase observed in the upper troposphere. In contrast, the direct radiative effect alone tends to reduce ice crystal concentrations (Fig. 5E). As shown in Fig. 5D, the indirect effect of dust aerosols on precipitation evolves over time: Before 25 April, the dusts reduce precipitation, whereas after 25 April, they substantially enhance precipitation. This shift is primarily attributed to the Bergeron process. Specifically, the heating effect of dust aerosols induces the evaporation of nearby cloud droplets, and the resulting water vapor subsequently condenses on ice crystal surfaces, leading to ice crystal decreases. Overall, the combined direct and indirect effects of dust lead to an increase in ice crystal number concentration (fig. S11B). Consequently, it can be found that during periods of weaker precipitation, the contributions of both the direct and indirect effects of dust to precipitation are relatively small. However, around 25 April, when precipitation begins to intensify, the direct radiative effect enhances precipitation earlier than the indirect effect, and together contribute positively to the peak precipitation observed on the 25th. After the 26th, the indirect effect of dust continues to increase precipitation, while the direct effect suppresses precipitation (Fig. 5, C and F). The indirect effect of dust enhances precipitation by 0.34% over 6 days under doubled dust concentrations, with a more pronounced increase (0.42%) during the final 3 days (Fig. 5F). When both the direct and indirect effects of dust are considered, the dust-induced precipitation increment is also the largest during the heaviest rainfall period (fig. S11C), which is consistent with the statistical results presented in Fig. 2.

### Competitive impact of dust and anthropogenic aerosols on precipitation

**Fig. 6.**
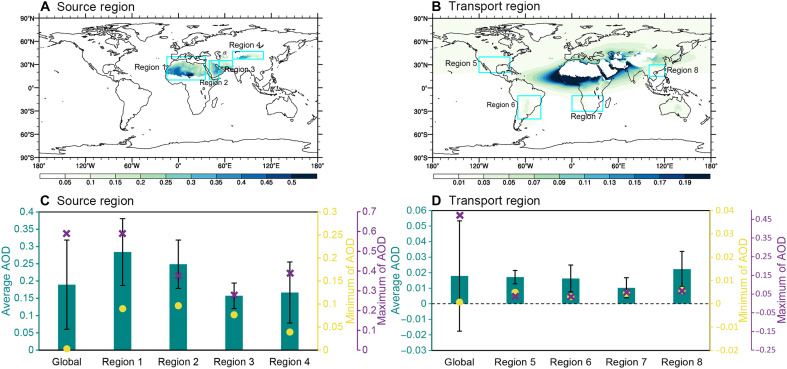
Global dust AOD distribution and regional extremes (2000–2019). (**A**) Spatial pattern of dust AOD in source regions. (**B**) Dust AOD distribution in transport regions. (**C**) Statistical distribution of dust AOD in source regions [cyan box in (A)], showing mean (bars), maximum (purple crosses), and minimum values (yellow dots). (**D**) Dust AOD statistics in transport regions [cyan box in (B)], showing mean (bars), maximum (purple crosses), and minimum values (yellow dots). Error bars (black lines) represent mean ± 1 SD.

To further understand the influence of coexisting dust and anthropogenic aerosols on precipitation from an observational perspective, we examined precipitation characteristics under varying levels of anthropogenic aerosol concentrations, both with and without dust aerosols, on a global scale. First, to establish a threshold for identifying dust aerosol presence, we analyzed the global distribution of dust aerosol optical depth (AOD) and its regional variations in extreme values. On the basis of the delineation of dust source and transport regions (fig. S1), the spatial distribution of global dust AOD across these regions is examined, along with the extreme values (maximum and minimum) in representative regions. The analysis reveals notably higher dust AOD in source regions relative to dust transport regions. Furthermore, dust AOD shows a distinct decrease with increasing distance from source regions within dust transport regions (Fig. 6, A and B).

Quantitative assessment of dust AOD extremes reveals distinct contrasts between source and transport regions. In dust source regions, minimum (maximum) dust AOD values span 0.003 to 0.096 (0.28 to 0.59) (Fig. 6C), whereas dust transport regions exhibit substantially lower ranges of 0.0008 to 0.0058 (0.02 to 0.49) (Fig. 6D). These findings indicate the considerable spatial heterogeneity in dust AOD at the global scale. Therefore, on the basis of the aforementioned extremal characteristics, a threshold of dust AOD ≥ 0.001 is adopted to define the dust presence, while values < 0.001 are classified as dust-free conditions, ensuring that the threshold is neither too small to be meaningful nor too large to overlook transport region samples with extremely low AOD in the statistical analysis.

Using the defined threshold, precipitation data are categorized by different anthropogenic AOD bins under both dust presence and dust-free conditions. These data are further classified by precipitation intensity, and occurrence frequencies are calculated for each precipitation intensity range. On the basis of stratified statistical analysis, the influence of dust aerosols is then isolated by comparing anthropogenic AOD-precipitation relationships between dust presence and dust-free conditions.

**Fig. 7.**
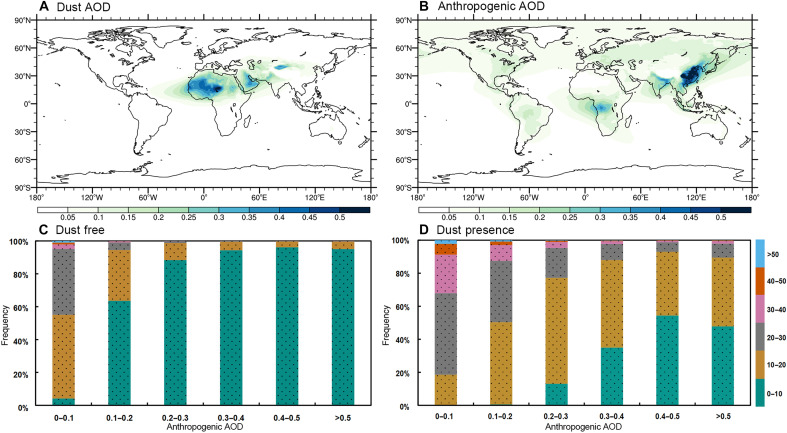
Contrasting effects of dust and anthropogenic aerosols on precipitation. (**A**) Spatial distribution of dust aerosols and (**B**) spatial distribution of anthropogenic aerosols during 2000–2019. (**C**) and (**D**) illustrate the influence of varying anthropogenic AOD levels on precipitation under different conditions: (C) dust free (dust AOD < 0.001) and (D) dust presence (dust AOD ≥ 0.001). The frequency distribution of precipitation across six ranges (0 to 10, 10 to 20, 20 to 30, 30 to 40, 40 to 50, and >50 mm) is analyzed for different anthropogenic AOD intervals (0 to 0.1, 0.1 to 0.2, 0.2 to 0.3, 0.3 to 0.4, 0.4 to 0.5, and >0.5) under both dust-free (C) and dust presence (D) conditions. The dotted bars in (C) and (D) represent values that are statistically significant at the 95% confidence level, as determined by bootstrap resampling.

Globally, regions with high dust AOD values are predominantly observed over major desert regions, including the Arabian Desert, Thar Desert, and Taklimakan Desert, with statistically significant increasing trends (Fig. 7A and fig. S12A; trend significance testing methodology is detailed in text S8). In contrast, although the Sahara Desert also exhibits high dust AOD, it has experienced a declining trend since the early 21st century. Anthropogenic aerosols, including black carbon, organic carbon, and sulfate, are predominantly concentrated in East Asia, South Asia, and central Africa, with a marked increase since the start of this century (Fig. 7B and fig. S12B); trend significance testing methodology is detailed in text S8. In addition, rising trends are evident in northwestern North America and northern South America, while substantial declines are observed in eastern North America and Europe. In key dust source regions such as the Arabian Desert, Iranian Plateau, and Taklimakan Desert, anthropogenic aerosols show modest growth. In contrast, the Sahara Desert exhibits a declining trend in anthropogenic aerosol levels.

Precipitation is further statistically analyzed across distinct aerosol conditions, comparing anthropogenic-dominated scenarios (dust AOD < 0.001) with mixed anthropogenic-dust environments (dust AOD ≥ 0.001), with spatiotemporal sampling variability rigorously evaluated (text S9 and fig. S13). Both temporal and spatial analyses demonstrate no systematic sampling bias between dust presence and dust-free conditions (Significance testing by bootstrap resampling is described in text S9 and fig. S13). As illustrated in Fig. 7C, in the absence of dust, when the anthropogenic AOD is below 0.1, precipitation predominantly falls within the 10- to 30-mm range, with notable occurrences in the 30- to 40-mm range and occasional events exceeding 50 mm. However, as the anthropogenic AOD increases, precipitation events below 10 mm become more frequent. Specifically, when the AOD surpasses 0.2, more than 80% of precipitation events fall below 10 mm, and this proportion exceeds 90% when the anthropogenic AOD exceeds 0.3. These findings indicate that in the absence of dust aerosols, higher concentrations of anthropogenic aerosols are associated with an increased likelihood of light precipitation. When dust aerosols are present, heavier precipitation occurs more frequently (Fig. 7D). Compared to conditions without dust, when the anthropogenic AOD is below 0.2, precipitation under 10 mm is almost absent, the proportion of precipitation between 10 and 20 mm decreases, and precipitation exceeding 30 mm increases substantially. However, as the concentration of anthropogenic aerosols rises, even with dust present, light precipitation gradually increases while heavier precipitation decreases. Once the anthropogenic AOD surpasses 0.3, precipitation above 40 mm no longer occurs.

Similar to the spatial heterogeneity observed in dust AOD (Fig. 7A), anthropogenic aerosol loading also exhibits pronounced regional variations (Fig. 7B). To examine aerosol-precipitation interactions more systematically, a refined binning approach (0 to 0.002 AOD increment) is implemented for the 0 to 0.02 concentration bin of anthropogenic aerosols (fig. S14). The analysis reveals that the dust presence remarkably amplifies heavier precipitation frequency, particularly when anthropogenic aerosol loading approaches the dust threshold (AOD = 0.001). This enhancement effect persists across varying background concentrations of anthropogenic aerosols, as consistently demonstrated through high-resolution AOD stratification analysis.

Overall, the statistical analysis results (Fig. 7, C and D, and fig. S14) align with the conclusions derived from the numerical simulations in Figs. 4 and 5. The mechanistic interpretation of statistical patterns in Fig. 7 (C and D) requires consideration of both dust aerosols’ ice-nucleating role (Fig. 4) and anthropogenic aerosols’ effects on precipitation. By comparing Coupled Model Intercomparison Project Phase 5 model simulations under Greenhouse Gases and anthropogenic aerosol forcing scenarios, we found that, on a long-term timescale, anthropogenic aerosols are the primary driver of the observed subtropical precipitation reduction during 1970–2005 (*30*). On the basis of this, we have investigated the influence of anthropogenic aerosols on short-term weather processes. It demonstrates that, on synoptic timescales, anthropogenic aerosols reduce total precipitation, with aerosol-radiation interactions and aerosol-cloud interactions jointly weakening light precipitation, thereby dominating the overall precipitation decline (*39*). The patterns in Fig. 7 (C and D) are attributed to the dominant influence of anthropogenic aerosols, which systematically increase light precipitation frequency while suppressing heavier precipitation events, irrespective of dust aerosol loading, thereby corroborating the mechanistic insights established in our previous studies (*30*, *39*). However, the critical distinction lies in the role of dust. Under identical anthropogenic aerosol concentrations, dust reduces light precipitation frequency while enhancing heavier precipitation, resulting in a net increase in total precipitation, as demonstrated by the mechanisms illustrated in Figs. 4 and 5. Mechanistically, acting as IN, dust particles promote the Bergeron effect, accelerating the growth of small raindrops and ultimately triggering precipitation through collision-coalescence (fig. S11). This is consistent with the findings from the numerical simulations (Figs. 4 and 5).

Our findings correspond with established understanding from air mass models and large-eddy simulation (LES) studies. Previous works have demonstrated that increased CCN concentrations reduce cloud droplet size while enhancing droplet number concentration (*40*, *41*), suppressing warm-rain processes through microphysical modifications. More recent LES modeling by Erfani *et al.* (*42*) showed that anthropogenic aerosols not only reduce droplet effective radius but also intensify evaporation, decreasing cloud optical depth. Calderón *et al.* (*43*) further revealed that high aerosol loadings enhance boundary layer turbulence, promoting droplet evaporation during subsidence, particularly near cloud base. Notably, when dust mixes with anthropogenic aerosols, the combined effects of spectral broadening and ice nucleation facilitate collision-coalescence and phase transitions, ultimately enhancing precipitation efficiency.

## DISCUSSION

This study examines global variations in dust events and their impacts on precipitation by integrating ground-based weather observations, reanalysis data, and the WRF model. The findings reveal that dust aerosols can enhance precipitation to varying extents worldwide, both in dust source and transport regions. Unlike prior qualitative studies on dust’s effects on precipitation, our research aims to quantify dust’s contribution under different scenarios. A key discovery is that in typical dust-affected regions, dust increases precipitation by affecting the cloud microphysics through amplifying the Bergeron process. Specifically, dust particles heat the surrounding atmosphere, inducing the evaporation of nearby cloud droplets and the subsequent release of water vapor (Fig. 5D). This vapor then condenses onto ice crystals (due to lower saturation vapor pressure over ice than that over liquid water), promoting their rapid growth (Fig. 5A). Through collision and coalescence, these ice crystals ultimately evolve into precipitation.

However, the influence of dust and anthropogenic aerosols on precipitation remains highly debated. The role of anthropogenic aerosols in modulating rainfall continues to be a contentious issue in climate science, with substantial evidence supporting both enhancement (*44*, *45*) and suppression (*30*, *46*–*48*) effects. The net effect depends on atmospheric conditions (*49*) and anthropogenic aerosol loading (*50*, *51*). Regional studies highlight this complexity, for instance, Menon *et al.* (*52*) demonstrated that anthropogenic aerosols increase precipitation in southeastern China but suppress it in northeastern China. Giorgi *et al.* (*53*) found that anthropogenic aerosols could potentially reduce precipitation across China; however, their study did not account for aerosol indirect effects, nor did it include organic carbon aerosols. Fan *et al.* (*49*) revealed a critical transition mechanism whereby increasing aerosol absorption efficiency can shift precipitation enhancement from indirect effects to suppression dominated by direct radiative forcing. Therefore, the impact of anthropogenic aerosols on precipitation exhibits remarkable regional variability and is also influenced by meteorological conditions.

Meanwhile, dust aerosols exhibit a dual influence on precipitation. On one hand, some studies have shown that dust can enhance precipitation by acting as IN, thereby intensifying convection—a finding consistent with our results. On the other hand, Min *et al.* (*54*) argued that the microphysical properties of dust aerosols can shift the precipitation spectrum from intense to weaker events, ultimately suppressing precipitation in regions like the Sahara Desert. In convective systems, dust aerosols can reduce the average size of cloud droplets, inhibiting the formation of warm rain (*55*). To validate this conclusion, further observations and additional regional simulations are required.

Aerosols are only one of three key factors influencing precipitation, alongside water vapor conditions and atmospheric uplift. The formation of precipitation is a complex physical process shaped by the interplay of these factors. In the broader context of climate studies, meteorological field discrepancies are not considered when assessing the impact of dust on precipitation. Dust influences precipitation through two contrasting mechanisms in meteorological systems. First, surface cooling caused by dust weakens the lower-tropospheric monsoon circulation, suppressing water vapor transport and rainfall (*37*). Conversely, adiabatic warming induced by dust enhances precipitation via the elevated heat pump (EHP) effect in the upper troposphere (*56*). On the basis of the EHP effect, the rising airflow over the desert is transported at high altitudes, forming a secondary circulation in surrounding regions and enhancing precipitation (*57*, *58*). While the precise meteorological mechanisms underlying dust-precipitation interactions remain unquantified, our composite analysis of contrasting circulation regimes reveals that dust-laden atmospheres tend to enhance precipitation compared to dust-free meteorological conditions (Fig. 2). Future work will address and expand on this aspect.

## MATERIALS AND METHODS

### Observational data and reanalysis datasets

In this study, surface site observations are sourced from the Integrated Surface Data (ISD; ISD v2.0, accessed November 2024) provided by the National Centers for Environmental Information. These observations include hourly records of weather phenomena such as freezing rain, dust events, and haze, collected from 35,000 global stations. The period from 1981 to 2010 serves as the standard climatological baseline to analyze the temporal patterns of dust anomalies in this study.

Bare soil areas are identified using average land use data from the International Geosphere-Biosphere Programme (IGBP) for the years 2001 to 2020. These data are derived from the Moderate-resolution Imaging Spectroradiometer. The identified bare soil areas are considered potential dust source regions, while regions outside these areas are classified as dust transport regions.

Global daily precipitation data from the CPC of the National Oceanic and Atmospheric Administration are analyzed for the period 1979–2023. These data are derived from 16,000 global stations, integrated with satellite observations, and processed using the optimal interpolation method to generate daily precipitation analysis products. The precipitation values are represented on a 0.5° latitude/longitude grid, providing area-averaged daily precipitation estimates for each grid.

AOD data from 2000–2019 are obtained from the Modern-Era Retrospective Analysis for Research and Applications, Version 2 (MERRA-2). These data are used to classify atmospheric conditions into two categories: dust presence (dust AOD ≥ 0.001) and dust-free (dust AOD < 0.001).

The Tropical Rainfall Measuring Mission (TRMM), a collaborative space mission by the NASA and Japan’s National Space Development Agency, provides precipitation data at a resolution of 0.5° × 0.5° for the period 2000–2019. These data are interpolated to align with the resolution of the MERRA-2 AOD data (0.5° × 0.625°) for statistical analysis. This analysis investigates the impact of dust aerosols on global precipitation under varying levels of anthropogenic aerosol influence.

### Definition of dust event

We analyzed data from the ISD database, encompassing 21,124 global sites with at least 1 month of observation records between 1979 and 2023. A dust event is defined as any day on which dust phenomena, including dust storms, blowing dust, or floating dust, are observed. Dust storms are characterized by visibility dropping below 1 km due to dense dust concentrations around the station. Blowing dust occurs when wind-driven particles in the vicinity of the station reduce visibility to between 1 and 10 km. Floating dust refers to widespread particulate matter suspended in the air near the observation station under low wind speed conditions. In addition, separate statistics are compiled for dust storms, floating dust, and blowing dust. If multiple types are observed on the same day, only the most severe type is recorded. For instance, if both a dust storm and floating dust occur on the same day, it is classified as a dust storm day. This study calculates the 45-year frequency of dust events for each station and examines annual patterns in dust event frequency on the global scale.

### Definition of accumulated precipitation

The CPC precipitation data are used to calculate accumulated precipitation. Precipitation influenced by dust events (prep_dust) refers to the total precipitation over the 7 days following a dust event, as identified using weather phenomena data from ISD. The difference between the global 7-day cumulative precipitation and prep_dust is calculated as prep_no_dust. The impact of dust events on precipitation is determined by the difference between prep_dust and prep_no_dust.

### Model description

The precipitation processes following two distinct dust storm processes are simulated using the WRF model to evaluate the impact of dust on precipitation in both a typical dust source region and a transport region (see fig. S7 for the specific simulation area). For the dust source region, we analyzed a dust event that occurred from 14 to 17 April 2020, in Northwest China (Dust_1). In addition, a dust event accompanied by precipitation, occurring from 22 to 27 April 2014, in North China (Dust_2), is selected as a representative case for the dust transport region. Both events are simulated using a two-layer nested domain structure. The model configurations are provided in table S2. To evaluate the robust impacts of doubled dust concentrations on cloud microphysics and precipitation, the sensitivity simulations using initial condition perturbation ensembles are performed (see text S6 and table S3).

### Significance testing

Composite meteorological field anomaly is assessed using the Monte Carlo field significance test, as described in text S5. A paired significance testing is used for ensemble perturbation experiment simulations, as detailed in text S7. The statistical relationship between aerosols and precipitation is assessed for significance testing using a bootstrap resampling method, outlined in text S9.

## Supplementary Materials

### This PDF file includes:

Supplementary Text S1 to S9 Figs. S1 to S15 Tables S1 to S3 References

## References

[1] X. Wang, O. Oenema, W. Hoogmoed, U. Perdok, D. Cai, Dust storm erosion and its impact on soil carbon and nitrogen losses in northern China. Catena 66, 221–227 (2006).

[2] D. Griffin, C. Kellogg, Dust storms and their impact on ocean and human health: Dust in Earth’s atmosphere. Ecohealth 1, 284–295 (2004).

[3] R. Duce, C. Unni, B. Ray, J. Prospero, J. Merrill, Long-range atmospheric transport of soil dust from Asia to the tropical North Pacific: Temporal variability. Science 209, 1522–1524 (1980). 17745962 10.1126/science.209.4464.1522

[4] T. Fairlie, D. Jacob, R. Park, The impact of transpacific transport of mineral dust in the United States. Atmos. Environ. 41, 1251–1266 (2007).

[5] J. Huang, P. Minnis, B. Chen, Z. Huang, Z. Liu, Q. Zhao, Y. Yi, J. K. Ayers, Long-range transport and vertical structure of Asian dust from CALIPSO and surface measurements during PACDEX. J. Geophys. Res. 113, D23212 (2008).

[6] A. Slingo, T. Ackerman, R. Allan, E. Kassianov, S. Mcfarlane, G. Robinson, J. Barnard, M. Miller, J. Harries, J. Russell, S. Dewitte, Observations of the impact of a major Saharan dust storm on the atmospheric radiation balance. Geophys. Res. Lett. 33, L24817 (2006).

[7] T. Y. Tanaka, Y. Kurosaki, M. Chiba, T. Matsumura, T. Nagai, A. Yamazaki, A. Uchiyama, N. Tsunematsu, K. Kai, Possible transcontinental dust transport from North Africa and the Middle East to East Asia. Atmos. Environ. 39, 3901–3909 (2005).

[8] R. Ding, J. Li, S. Wang, F. Ren, Decadal change of the spring dust storm in northwest China and the associated atmospheric circulation. Geophys. Res. Lett. 32, L02808 (2005).

[9] T. Shao, Y. Liu, Z. Tan, D. Li, M. Luo, R. Luo, Characteristics and a mechanism of dust weather in northern China. Climate Dynam. 61, 1591–1606 (2022).

[10] C. Zhou, Y. Liu, Q. Zhu, Q. He, T. Zhao, F. Yang, W. Huo, X. Yang, A. Mamtimin, In situ observation of warm atmospheric layer and the heat contribution of suspended dust over the Tarim Basin. Atmos. Chem. Phys. 22, 5195–5207 (2022).

[11] Z. Liu, A. Omar, M. Vaughan, J. Hair, C. Kittaka, Y. Hu, K. Powell, C. Trepte, D. Winker, C. Hostetler, R. Ferrare, R. Pierce, CALIPSO lidar observations of the optical properties of Saharan dust: A case study of long-range transport. J. Geophys. Res. 113, D07207 (2008).

[12] Y. Liu, J. Huang, G. Shi, T. Takamura, P. Khatri, J. Bi, J. Shi, T. Wang, X. Wang, B. Zhang, Aerosol optical properties and radiative effect determined from sky-radiometer over Loess Plateau of Northwest China. Atmos. Chem. Phys. 11, 11455–11463 (2011).

[13] Y. Liu, R. Jia, T. Dai, Y. Xie, G. Shi, A review of aerosol optical properties and radiative effects. J. Meteor. Res. 28, 1003–1028 (2014).

[14] J. Huang, Q. Fu, J. Su, Q. Tang, P. Minnis, Y. Hu, Y. Yi, Q. Zhao, Taklimakan dust aerosol radiative heating derived from CALIPSO observations using the Fu-Liou radiation model with CERES constraints. Atmos. Chem. Phys. 9, 4011–4021 (2009).

[15] J. Li, B. Carlson, Y. Yung, D. Lv, J. Hansen, J. Penner, H. Liao, V. Ramaswamy, R. Kahn, P. Zhang, O. Dubovik, A. Ding, A. Lacis, L. Zhang, Y. Dong, Scattering and absorbing aerosols in the climate system. Nat. Rev. Earth Environ. 3, 363–379 (2022).

[16] Y. Sun, C. Zhao, Distinct impacts on precipitation by aerosol radiative effect over three different megacity regions of eastern China. Atmos. Chem. Phys. 21, 16555–16574 (2021).

[17] C. Zhao, Y. Yang, H. Fan, J. Huang, Y. Fu, X. Zhang, S. Kang, Z. Cong, H. Letu, M. Menenti, Aerosol characteristics and impacts on weather and climate over the Tibetan Plateau. Natl. Sci. Rev. 7, 492–495 (2020). 34692068 10.1093/nsr/nwz184 PMC8288843

[18] A. Li, C. Shi, S. Yin, N. Li, H. Letu, G. Shi, Variation of surface solar radiation components from 2016 to 2020 in China: Perspective from geostationary satellite observation with a high spatiotemporal resolution. Sci. Total Environ. 954, 176264 (2024). 39284448 10.1016/j.scitotenv.2024.176264

[19] J. Penner, D. Hegg, R. Leaitch, Unraveling the role of aerosols in climate change. Environ. Sci. Technol. 35, 332–340 (2001). 10.1021/es0124414 11506021

[20] S. Twomey, The influence of pollution on the shortwave albedo of clouds. J. Atmos. Sci. 34, 1149–1152 (1977).

[21] D. Rosenfeld, S. Sherwood, R. Wood, L. Donner, Climate effects of aerosol-cloud interactions. Science 343, 379–380 (2014). 24458631 10.1126/science.1247490

[22] C. Zhao, Y. Lin, F. Wu, Y. Wang, Z. Li, D. Rosenfeld, Y. Wang, Enlarging rainfall area of tropical cyclones by atmospheric aerosols. Geophys. Res. Lett. 45, 8604–8611 (2018).

[23] C. Zhao, Y. Qiu, X. Dong, Z. Wang, Y. Peng, B. Li, Z. Wu, Y. Wang, Negative aerosol-cloud r_e_ relationship from aircraft observations over Hebei, China. Earth Space Sci. 5, 19–29 (2018).

[24] C. Zhao, T. J. Garrett, Effects of Arctic haze on surface cloud radiative forcing. Geophys. Res. Lett. 42, 557–564 (2015).

[25] Y. Liu, Q. Zhu, J. Huang, S. Hua, R. Jia, Impact of dust-polluted convective clouds over the Tibetan Plateau on downstream precipitation. Atmos. Environ. 209, 67–77 (2019).

[26] Y. Liu, S. Hua, R. Jia, J. Huang, Effect of aerosols on the ice cloud properties over the Tibetan Plateau. J. Geophys. Res. Atmos. 124, 9594–9608 (2019).

[27] Y. Liu, Q. Zhu, S. Hua, A. Khan, T. Dai, Y. Cheng, Tibetan Plateau driven impact of Taklimakan dust on northern rainfall. Atmos. Environ. 234, 117583 (2020).

[28] Y. Liu, Y. Li, J. Huang, Q. Zhu, S. Wang, Attribution of the Tibetan Plateau to northern drought. Natl. Sci. Rev. 7, 489–492 (2020). 34692067 10.1093/nsr/nwz191 PMC8288960

[29] Y. Liu, J. Huang, T. Wang, J. Li, H. Yan, Y. He, Aerosol-cloud interactions over the Tibetan Plateau: An overview. Earth Sci. Rev. 234, 104216 (2022).

[30] Y. Liu, T. Shao, S. Hua, Q. Zhu, R. Luo, Association of anthropogenic aerosols with subtropical drought in East Asia. Int. J. Climatol. 40, 3500–3513 (2020).

[31] R. Jia, Y. Liu, S. Hua, Q. Zhu, T. Shao, Estimation of the aerosol radiative effect over the Tibetan Plateau based on the latest CALIPSO product. J. Meteorol. Res. 32, 707–722 (2018).

[32] A. McComiskey, G. Feingold, The scale problem in quantifying aerosol indirect effects. Atmos. Chem. Phys. 12, 1031–1049 (2012).

[33] J. Fan, Y. Wang, D. Rosenfeld, X. Liu, Review of aerosol-cloud interactions: Mechanisms, significance and challenges. J. Atmos. Sci. 73, 4221–4252 (2016).

[34] F. Glassmeier, F. Hoffmann, J. Johnson, T. Yamaguchi, K. Carslaw, G. Feingold, Aerosol-cloud-climate cooling overestimated by ship-track data. Science 371, 485–489 (2021). 33510021 10.1126/science.abd3980

[35] A. Arola, A. Lipponen, P. Kolmonen, T. H. Virtanen, N. Bellouin, D. P. Grosvenor, E. Gryspeerdt, J. Quaas, H. Kokkola, Aerosol effects on clouds are concealed by natural cloud heterogeneity and satellite retrieval errors. Nat. Commun. 13, 7357 (2022). 36446763 10.1038/s41467-022-34948-5 PMC9708656

[36] J. Huang, P. Minnis, H. Yan, Y. Yi, B. Chen, L. Zhang, J. K. Ayers, Dust aerosol effect on semi-arid climate over Northwest China detected from A-Train satellite measurements. Atmos. Chem. Phys. 10, 6863–6872 (2010).

[37] R. Luo, Y. Liu, M. Luo, D. Li, Z. Tan, T. Shao, A. Khan, Dust effects on mixed-phase clouds and precipitation during a super dust storm over northern China. Atmos. Environ. 313, 120081 (2023).

[38] R. Sequeira, On the large-scale impact of arid dust on precipitation chemistry of the continental northern hemisphere. Atmos. Environ. Part A Gen. Top. 27, 1553–1565 (1993).

[39] T. Shao, Y. Liu, R. Wang, Q. Zhu, Z. Tan, R. Luo, Role of anthropogenic aerosols in affecting different-grade precipitation over eastern China: A case study. Sci. Total Environ. 807, 150886 (2022). 34634341 10.1016/j.scitotenv.2021.150886

[40] U. Dusek, M. Frank, P. Hildebrandt, L. Curtius, J. Schneider, S. Walter, D. Chand, F. Drewnick, S. Hings, D. Jung, S. Borrmann, M. O. Andreae, Size matters more than chemistry for cloud-nucleating ability of aerosol particles. Science 312, 1375–1378 (2006). 16741120 10.1126/science.1125261

[41] H. Xue, G. Feingold, B. Stevens, Aerosol effects on clouds, precipitation, and the organization of shallow cumulus convection. J. Atmos. Sci. 65, 392–406 (2008).

[42] E. Erfani, P. Blossey, R. Wood, J. Mohrmann, S. J. Doherty, M. Wyant, K.-T. O., Simulating aerosol lifecycle impacts on the subtropical stratocumulus-to-cumulus transition using large-eddy simulations. J. Geophys. Res. Atmos. 127, e2022JD037258 (2022).

[43] S. M. Calderón, J. Tonttila, A. Buchholz, J. Joutsensaari, M. Komppula, A. Leskinen, L. Hao, D. Moisseev, I. Pullinen, P. Tiitta, J. Xu, A. Virtanen, H. Kokkola, S. Romakkaniemi, Aerosol–stratocumulus interactions: Towards a better process understanding using closures between observations and large eddy simulations. Atmos. Chem. Phys. 22, 12417–12441 (2022).

[44] A. Khain, D. Rosenfeld, A. A. Pokrovsky, Aerosol impact on the dynamics and microphysics of deep convective clouds. Q. J. Roy. Meteorol. Soc. 131, 2639–2663 (2005).

[45] J. Fan, D. Rosenfeld, Y. Zhang, S. E. Giangrande, Z. Li, L. A. T. Machado, S. T. Martin, Y. Yang, J. Wang, P. Artaxo, H. M. J. Barbosa, R. Braga, J. M. Comstock, Z. Feng, W. Gao, H. B. Gomes, F. Mei, C. Pöhlker, M. L. Pöhlker, U. Pöschl, R. A. F. D. Souza, Substantial convection and precipitation enhancements by ultrafine aerosol particles. Science 359, 411–418 (2018). 29371462 10.1126/science.aan8461

[46] Y. Qian, D. Gong, J. Fan, L. R. Leung, R. Bennartz, D. Chen, W. Wang, Heavy pollution suppresses light rain in China: Observations and modeling. J. Geophys. Res. 114, D00K02 (2009).

[47] X. Liu, X. Xie, Z. Yin, C. Liu, A. Gettelman, A modeling study of the effects of aerosols on clouds and precipitation over East Asia. Theor. Appl. Climatol. 106, 343–354 (2011).

[48] J. Guo, M. Deng, S. S. Lee, F. Wang, Z. Li, P. Zhai, H. Liu, W. Lv, W. Yao, X. Li, Delaying precipitation and lightning by air pollution over the Pearl River Delta. Part I: Observational analyses. J. Geophys. Res. Atmos. 121, 6472–6488 (2016).

[49] J. Fan, R. Zhang, W.-K. Tao, K. I. Mohr, Effects of aerosol optical properties on deep convective clouds and radiative forcing. J. Geophys. Res. 113, D08209 (2008).

[50] D. Rosenfeld, U. Lohmann, G. B. Raga, C. D. O’Dowd, M. Kulmala, S. Fuzzi, A. Reissell, M. O. Andreae, Flood or drought: How do aerosols affect precipitation? Science 321, 1309–1313 (2008). 18772428 10.1126/science.1160606

[51] I. Koren, Y. J. Kaufman, L. A. Remer, J. V. Martins, Measurement of the effect of Amazon smoke on inhibition of cloud formation. Science 303, 1342–1345 (2004). 14988557 10.1126/science.1089424

[52] S. Menon, J. Hansen, L. Nazarenko, Y. Luo, Climate effects of black carbon aerosols in China and India. Science 297, 2250–2253 (2002). 12351786 10.1126/science.1075159

[53] F. Giorgi, X. Bi, Y. Qian, Direct radiative forcing and regional climatic effects of anthropogenic aerosols over East Asia: A regional coupled climate-chemistry/aerosol model study. J. Geophys. Res. 107, 4439 (2002).

[54] Q. Min, R. Li, B. Lin, E. Joseph, S. Wang, Y. Hu, V. Morris, F. Chang, Evidence of mineral dust altering cloud microphysics and precipitation. Atmos. Chem. Phys. 19, 3223–3231 (2009).

[55] I. Koren, Y. J. Kaufman, D. Rosenfeld, L. A. Remer, Y. Rudich, Aerosol invigoration and restructuring of Atlantic convective clouds. Geophys. Res. Lett. 32, L14808 (2005).

[56] K. M. Lau, M. K. Kim, K. M. Kim, Asian summer monsoon anomalies induced by aerosol direct forcing: The role of the Tibetan Plateau. Clim. Dyn. 26, 855–864 (2006).

[57] V. Vinoj, P. J. Rasch, H. Wang, J. Yoon, P. Ma, K. Landu, B. Singh, Short-term modulation of Indian summer monsoon rainfall by West Asian dust. Nat. Geosci. 7, 308–313 (2014).

[58] F. Solmon, V. S. Nair, M. Mallet, Increasing Arabian dust activity and the Indian summer monsoon. Atmos. Chem. Phys. 15, 8051–8064 (2015).

[59] P. Knippertz, M. C. Todd, Mineral dust aerosols over the Sahara: Meteorological controls on emission and transport and implications for modeling. Rev. Geophys. 50, RG1007 (2012).

[60] A. Prein, P. Mooney, J. Done, The multi-scale interactions of atmospheric phenomenon in mean and extreme precipitation. Earth’s Future 11, e2023EF003534 (2023).

[61] B. Dieppois, A. Diedhiou, A. Durand, M. Fournier, N. Massei, D. Sebag, Y. Xue, B. Fontaine, Quasi-decadal signals of Sahel rainfall and West African monsoon since the mid-twentieth century. J. Geophys. Res. Atmos. 118, 12587–12599 (2013).

[62] R. E. Livezey, W. Y. Chen, Statistical field significance and its determination by Monte Carlo technique. Mon. Weather Rev. 111, 46–59 (1983).

[63] D. S. Wilks, “The stippling shows statistically significant grid points”: How research results are routinely overstated and overinterpreted, and what to do about it. Bull. Am. Meteorol. Soc. 97, 2263–2273 (2016).

[64] D. R. Stratman, N. Yussouf, C. A. Kerr, B. C. Matilla, J. R. Lawson, Y. Wang, Testing stochastic and perturbed parameter methods in an experimental 1-km warn-on-forecast system using NSSL’s phased-array radar observations. Mon. Weather Rev. 152, 433–454 (2024).

[65] S. Wang, J. Min, X. Li, X. Qiao, An atmospheric instability perturbation approach for ensemble forecasts and its application in heavy rain cases. J. Adv. Model. Earth Syst. 17, e2024MS004556 (2025).

[66] M. Mudelsee, Statistical analysis in climate research. Comput. Geosci. 27, 371–373 (2001).

[67] S. N. Lahiri, Theoretical comparisons of block bootstrap methods. Ann. Stat. 27, 386–404 (1999).

[68] P. Hall, J. L. Horowitz, B.-Y. Jing, On blocking rules for the bootstrap with dependent data. Biometrika 82, 561–574 (1995).

[69] G. Thompson, T. Eidhammer, A study of aerosol impacts on clouds and precipitation development in a large winter cyclone. J. Atmos. Sci. 71, 3636–3658 (2014).

[70] J. Kain, J. Fritsch, A one-dimensional entraining/detraining plume model and its application in convective parameterization. J. Atmos. Sci. 47, 2784–2802 (1990).

[71] J. Kain, The Kain-Fritsch convective parameterization: An update. J. Appl. Meteorol. 43, 170–181 (2004).

[72] E. Mlawer, S. Taubman, P. Brown, M. J. Iacono, S. A. Clough, Radiative transfer for inhomogeneous atmospheres: RRTM, a validated correlated-k model for the longwave. J. Geophys. Res. 102, 16663–16682 (1997).

[73] M. Iacono, E. Mlawer, S. Clough, J. Morcrette, Impact of an improved longwave radiation model, RRTM, on the energy budget and thermodynamic properties of the NCAR community climate model, CCM3. J. Geophys. Res. 105, 14873–14890 (2000).

[74] S. Hong, Y. Noh, J. Dudhia, A new vertical diffusion package with an explicit treatment of entrainment processes. Mon. Weather Rev. 134, 2318–2341 (2006).

[75] J. Dudhia, A multi-layer soil temperature model for MM5. *Proc. 6th PSU/NCAR Mesoscale Model Users’ Workshop* (1996).

