# Supplementary material for: Dust storms: Hidden drivers of extreme rainfall and global precipitation shifts
Source: Sci Adv. 2026 Apr 29;12(18):eadw6864. doi: 10.1126/sciadv.adw6864 (PMC13127582; doi:10.1126/sciadv.adw6864)
Supplement: Supplementary file 1 — Supplementary Text S1 to S9 Figs. S1 to S15 Tables S1 to S3 References [file sciadv.adw6864_sm.pdf]

Science Advances
AAAS

Supplementary Materials for

Dust storms: Hidden drivers of extreme rainfall and global precipitation shifts

Yuzhi Liu *et al.*

Corresponding author: Jianping Huang, hjp@lzu.edu.cn

Sci. Adv. **12**, eadw6864 (2026)
DOI: 10.1126/sciadv.adw6864

This PDF file includes:

Supplementary Text S1 to S9
Figs. S1 to S15
Tables S1 to S3
References

Supplementary Text

S1. Definition of Dust Source Regions and Dust Transport Regions

In this study, the identified bare soil areas are considered potential dust source regions, while regions outside these areas are classified as dust transport regions. Bare soil areas are identified using average land use data from the International Geosphere-Biosphere Programme (IGBP) for the years 2001 to 2020. This data is derived from the Moderate-resolution Imaging Spectroradiometer (MODIS). The fig. S1 presents the global distribution of dust source regions and transport regions.

S2. Methodology of Calculating the 7-day Cumulative Precipitation Difference

(1) Identification of Dust Events. Daily weather phenomenon data from the Integrated Surface Database (ISD) station observations (1979–2023) are used to identify stations where dust events occurred.

(2) Matching Dust Events with Precipitation Data. The station-observed dust event data are matched with daily accumulated precipitation data from the Climate Prediction Center (CPC). For instance, if Station A recorded a dust event on date X, its latitude and longitude are compared with CPC grid points. When the spatial discrepancy between the station and the nearest CPC grid point is <0.25°, the CPC grid's daily precipitation value is assigned to Station A. Subsequently, the 7-day accumulated precipitation following each dust event (hereafter referred to as dust-event 7-day precipitation) is computed.

(3) Calculation of Dust-free Precipitation. Using CPC data, the 7-day accumulated precipitation for each global grid point is computed as the total 7-day accumulated precipitation. The dust-free 7-day accumulated precipitation is then derived by subtracting the dust-event precipitation (from step 2) from the total 7-day accumulated precipitation at each CPC grid point.

(4) Estimation of Dust's Impact on Precipitation. The influence of dust aerosols on 7-day accumulated precipitation is assessed by calculating the difference between dust-event precipitation (derived in step 2) and dust-free precipitation (derived in step 3).

This approach provides an approximate measure of dust's effect on precipitation. Following the methodology described above, the spatial distribution of differences in 7-day accumulated precipitation between dust events and dust-free conditions is calculated.

S3. Calculation Method for the Precipitation Increase

Based on the above approach of calculating the 7-day cumulative precipitation difference, the globally averaged monthly 7-day accumulated precipitation is calculated separately under dust events and dust-free conditions.

This process generates two time series: (1) "monthly 7-day accumulated precipitation under dust event conditions" and (2) "monthly 7-day accumulated precipitation under dust-free conditions". Subsequently, the maximum, minimum, median, 25th percentile, and 75th percentile values are extracted from each time series. By analyzing these statistical metrics, we obtained information on the intensity and variability of the monthly mean 7-day accumulated precipitation under dust events and dust-free conditions from 1979 to 2023. Statistical results indicate that during the study period, the characteristic values (maximum, minimum, median, 25th, and 75th percentiles) of 7-day accumulated precipitation under dust event conditions are consistently higher than those under dust-free conditions across the globe.

On a global scale, the maximum value in the monthly 7-day accumulated precipitation time series under dust-free conditions is 14.2 mm, while under dust conditions, it reaches 23.8 mm—a difference of 9.6 mm. This 9.6 mm represents the maximum increase in monthly mean 7-day accumulated precipitation under dust events conditions at the global scale, suggesting that dust aerosols may enhance precipitation by up to 9.6 mm under extreme conditions.

S4. Composite Analysis of Large-Scale Circulation Patterns

Composite Analysis (also referred to as conditional sampling) is widely used to investigate the response characteristics of circulation fields under specific climatic or weather conditions (*59–61*). In this study, composite analysis is employed to examine the influence of meteorological fields (F) on precipitation during dust events (I). As the first step in the analysis, positive and negative events are defined. A positive event of I is defined when multiple stations worldwide concurrently observe dust events on the same day. A negative event of I is defined when multiple stations globally report no dust event observations on the same day. To determine the most reasonable threshold for the number of stations to select positive and negative events, we established tiered detection thresholds for both event types. For positive events, we set the screening thresholds to no fewer than 100, 150, 200, 250, and 300 stations based on the number of global stations observing dust on the same day. For negative events, the selection criteria require ⩾20,900, 20,950, 21,000, 21,050, or 21,100 stations globally reporting no dust detection on the same day. This stratified sampling generates distinct event counts for each threshold during 1979–2023, as stricter criteria naturally yield fewer qualifying days. While screening samples, it is required that as many stations as possible observe the dust events simultaneously to ensure their widespread impact on a global scale. However, it is very difficult to require thousands of stations worldwide to observe the dust events on the same day. Even so, we selected the samples with the maximum number of stations simultaneously observing and not observing dust events for composite analysis. Finally, to optimize the trade-off between noise reduction and sample size, we selected dates featuring simultaneous dust events at least 250 stations globally (hereafter "dust-active days"), yielding 161 qualifying days. For these days, composite analyses of geopotential height, wind, and humidity fields at 850 hPa and 500 hPa levels are performed by using National Centers for Environmental Prediction (NCEP) reanalysis data. Similarly, we selected dates with over 21,100 stations reporting no dust events (hereafter termed "dust-inactive days"), totaling 98 days, and conducted analogous analyses for the same atmospheric variables at the same pressure levels. Based on the global classification of dust-active and dust-inactive days, anomalies in geopotential height and wind fields at 850 hPa and 500 hPa, along with corresponding relative humidity anomalies are analyzed. The "anomalies" represent deviations of dust-active days relative to dust-inactive days, where positive (negative) values indicate higher (lower) magnitudes in dust-active conditions for each variable.

S5. Significance Testing for Meteorological Composite Fields

While pointwise t-tests are widely used for meteorological anomaly analysis, they assume spatial independence—an assumption violated by inherent spatial correlations in real fields. This can produce false positives with large sample sizes (*62*). We therefore implemented Monte Carlo field significance testing (*63*) as follows:

(1) Sample classification. Dust-active days (>250 global stations reporting dust) and dust-inactive days (>21,100 stations reporting no dust) yielded 161 high- and 98 low-frequency samples.

(2) Random field generation. Pooling all 259 samples, we generated 1,000 random permutations by shuffling indices (0–258). For each permutation, the first 161 samples were labeled "high-frequency", the rest were labeled "low-frequency", preserving spatial covariance.

(3) Pointwise testing (real fields). Two-sample t-tests ($p \leqslant 0.05$) were applied to real high/low-frequency fields (wind, geopotential height, RH) at each grid point.

(4) Monte Carlo simulation. Step 3 was repeated for all 1,000 random field pairs, creating a null p-value distribution (n = 1,000 per grid point).

(5) Thresholding. The 5th percentile of each grid point's null p-value distribution defined the local field significance threshold. Only grid points passing both pointwise ($p \leqslant 0.05$) and field-wide ($p \leqslant 0.05$) tests were deemed significant (black dots in Fig. 3, fig. S6).

S6. Numerical Experiments and Perturbation Methodology

(1) Aerosol Prescription and Experimental Design. In the Weather Research and Forecasting (WRF) model simulations, aerosol concentrations are prescribed. For the control experiment (CTL), dust concentrations are sourced from the 2001–2007 climatological dust background as modeled by the Goddard Chemistry Aerosol Radiation and Transport (GOCART) model. The Thompson aerosol-aware microphysics scheme is utilized to differentiate between hygroscopic aerosols (such as organic carbon, sulfate, and sea salt) and non-hygroscopic ice-nucleating aerosols (such as dust). A comparison between the dust concentrations in Modern-Era Retrospective analysis for Research and Applications, Version 2 (MERRA-2, considered as observational truth) and the CTL experiment (fig. S15) reveals that actual dust concentrations during dust events can exceed the model's prescribed values by up to a factor of 10. To evaluate the potential impact of this underestimation, sensitivity simulations are conducted with doubled (2×) dust concentrations to explore their effects on cloud microphysics and precipitation in this study.

(2) Ensemble Perturbation Approach for Uncertainty Quantification. To quantify uncertainties arising from initial conditions, an ensemble approach was employed by generating perturbed initial fields through stochastic perturbations (*64*). This allows for a systematic evaluation of initial-condition errors (*65*). The RANDOMCV module within the WRF Data Assimilation (WRFDA) system was used to introduce physically consistent perturbations across multiple variables. Unlike white-noise perturbations, this method preserves the background error covariance structure, adjusts interrelated meteorological variables (such as temperature, humidity, and wind fields) synchronously, and maintains dynamic and thermodynamic balance.

(3) Perturbation Ensemble Configuration. The perturbation ensemble is divided into two main experimental groups: the dust source region experiment (Dust_1), which includes only the aerosol indirect effect (AIE), and the dust transport region experiment (Dust_2), which encompasses both direct radiative effect (DRE) and AIE. Since the Thompson scheme does not simulate DRE explicitly, the DRE is derived as the difference between the total dust effect (DRE + AIE) and the AIE-only effect. In this study, 10 initial-condition perturbations are conducted for each scenario (1× and 2× dust concentrations), resulting in 6 major experimental groups and a total of 60 ensemble members. To limit perturbation magnitudes, a 10% perturbation was applied to the unbalanced surface pressure variance in the initial fields, which automatically triggered dynamically consistent adjustments in related variables (such as geopotential height, temperature, dry air mass, and pressure) while maintaining physical coherence.

(4) Ensemble Consistency and Dust Radiative Effects. By fixing the random seed parameters (seed_array1, seed_array2), identical stochastic perturbations are ensured between the 1× and 2× dust experiments, maintaining a one-to-one correspondence across ensemble members. For the Dust_1 case, paired 10-member ensemble experiments are conducted under 1× (CTL) and 2× (sensitivity) dust conditions, applying identical perturbations to corresponding members. The Dust_2 experiments followed a similar methodology but incorporated both DRE and AIE aerosol effects.

Model configurations and experimental designs are detailed in Supplementary tables S2–S3.

S7. Paired Significance Testing for Ensemble Perturbation Experiments

To account for potential errors arising from spatiotemporal autocorrelation, we performed paired t-tests (*66*) on the outcomes of initial field random perturbation experiments. The WRFDA system generated 10 perturbed ensemble members with random yet mutually independent perturbations in the initial fields, enabling each member's simulation results to be regarded as an independent experiment. For each ensemble member, we calculated the differences in meteorological variables between the 2× dust and 1× dust scenarios using the following equations:

$$D(i) = P_{dust}(i) - P_{ctrl}(i) \quad (1)$$

$$\bar{D} = \sum_{i=0}^{N} D(i) / N \quad (2)$$

$$\text{t} = \frac{\bar{D}}{S_D/\sqrt{N}} \quad (3)$$

where i is the ensemble member index. $D(i)$ represents the difference in physical variables (e.g., ice crystal number concentration, precipitation amount, and ice mixing ratio) between the 2 × dust (P_{dust}) and 1 × dust (P_{ctrl}) experiments for the *i-th* ensemble member. $\bar{D}$ denotes the mean difference across all ensemble members, N is the total number of ensemble members. S_D is the standard deviation of the differences $D(i)$.

S8. Significance Testing for Aerosol Optical Depth (AOD) Trends

Given the pronounced autocorrelation structures in the dust and anthropogenic aerosol time series, the construction of their randomized fields requires preservation of temporal autocorrelation. To address this, we employed the block bootstrap method to generate surrogate samples. The implementation steps are as follows:

(1) Block Length Selection. According to Lahiri (*67*), the optimal block length (L) for a time series of length n scales proportionally to $n^{1/3}$. Empirical studies suggest that blocks of 3–5 years effectively balance sample independence and temporal dependence (*68*). For our 20-year dataset, we set $L = 4$ years after evaluating computational efficiency and statistical robustness.

(2) Random Field Generation and Trend Analysis. The same resampling framework as in text S5 is used to construct 1,000 randomized fields each for dust and anthropogenic aerosols. Linear regression coefficients and their associated p-values are computed for both the observed and surrogate fields.

(3) Significance Threshold. The 5th percentile of *p*-values across all surrogate fields defined the significance threshold ($\alpha = 0.05$). Grid points in the real aerosol field with p-values below this threshold are deemed statistically significant at the 95% confidence level.

S9. Bootstrap Resampling Methodology for Aerosol-Precipitation Relation Analysis

The Bootstrap method provides a nonparametric approach for statistical inference without requiring assumptions about the underlying data distribution. Through repeated resampling with replacement from the original datasets, the method constructs an empirical distribution of the test statistic, allowing robust estimation of confidence intervals and significance levels. Compared to conventional parametric tests, the Bootstrap approach demonstrates superior reliability when analyzing climate data with strong non-stationarity or complex correlation structures. This analysis aims to verify whether there is a significant difference between the precipitation influenced by dust and anthropogenic aerosols (hereinafter referred to as “actual precipitation”) and randomly selected precipitation (hereinafter referred to as “random precipitation”) within the same temporal and spatial ranges. The procedure consists of the following steps:

(1) Spatiotemporal alignment of precipitation data. Global precipitation data are partitioned into 10 °×10 ° grid cells. For each temporal observation, precipitation events influenced by dust or anthropogenic aerosols are identified. Corresponding grid locations are determined using latitude and longitude coordinates, with all precipitation records within matching grids extracted to maintain spatiotemporal consistency between actual and randomly sampled precipitation events.

(2) Bootstrap resampling implementation. The selected precipitation samples are converted into a one-dimensional array, and 1,000 random resamplings with replacement are performed to generate a series of random precipitation fields. This resampling procedure is executed independently for each time step and AOD bin category to ensure comprehensive sampling of the statistical distribution.

(3) Frequency distribution computation. Precipitation intensities are categorized into discrete bins. For each bootstrap-generated precipitation field, we calculate the occurrence frequency within each intensity bin to characterize the probability distribution of random precipitation events across different intensity ranges.

(4) Statistical significance test. The frequencies of random precipitation in each bin are sorted, and the 2.5th and 97.5th percentiles are extracted as the lower and upper bounds of the 95% confidence interval. If the frequency of actual precipitation in a given bin falls outside this range, the difference is considered statistically significant at the 95% confidence level; otherwise, the result is deemed non-significant.

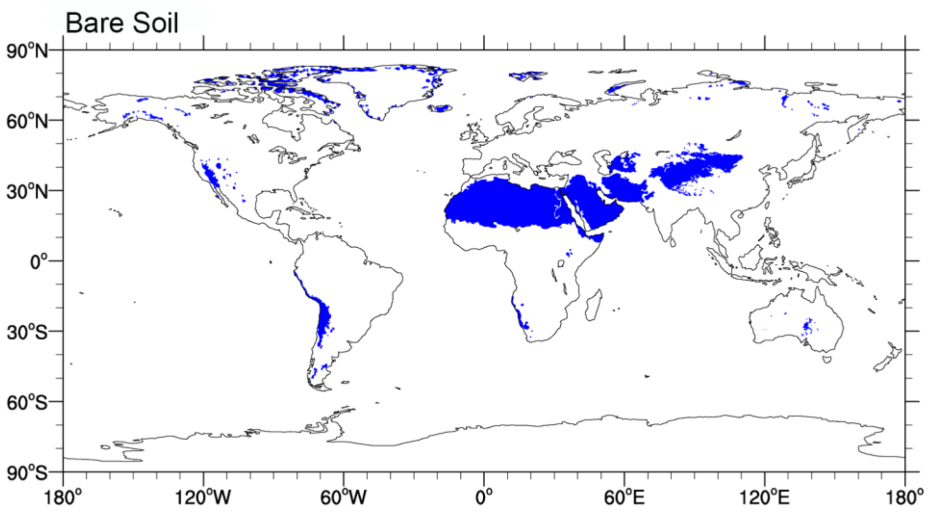

fig. S1. Geographic distribution of global dust source regions (shaded) and transport regions (unshaded).

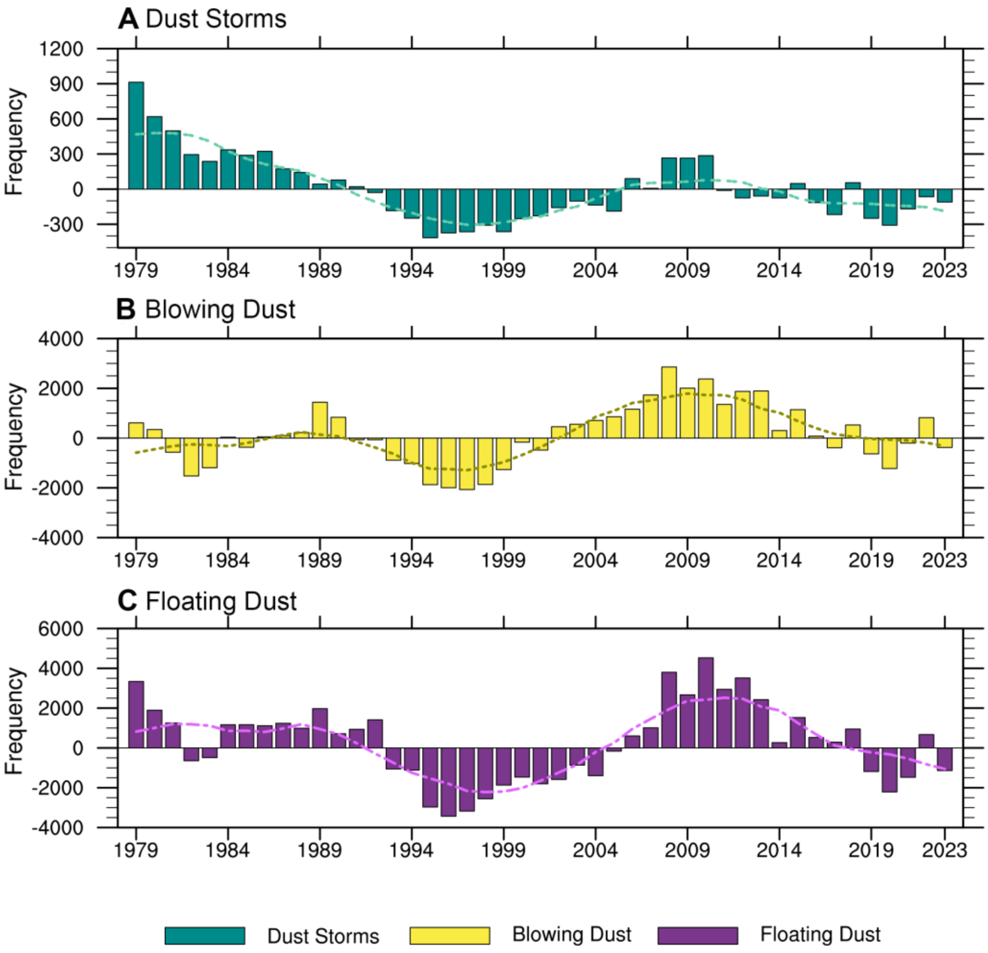

fig. S2. Time series of frequency anomalies for dust events in global dust source regions from 1979 to 2023. (**A**) Dust storms, (**B**) blowing dust, and (**C**) floating dust (units: occurrences). The cyan, yellow, and purple curves in (A), (B), and (C) represent 9-point smoothed trends.

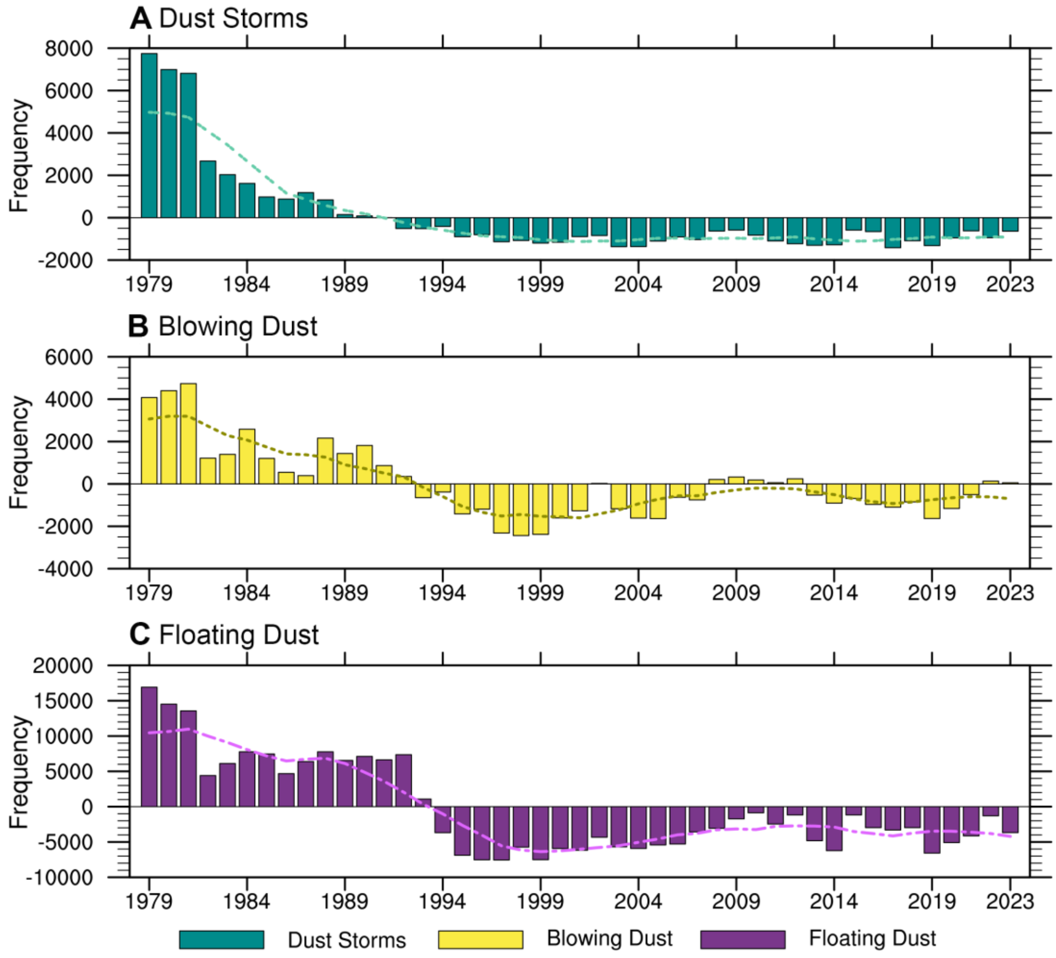

fig. S3. Time series of frequency anomalies for dust events in the global dust transport regions from 1979 to 2023. (**A**) dust storms, (**B**) blowing dust, and (**C**) floating dust (units: occurrences). The cyan, yellow, and purple curves in (A), (B), and (C) represent 9-point smoothed trend.

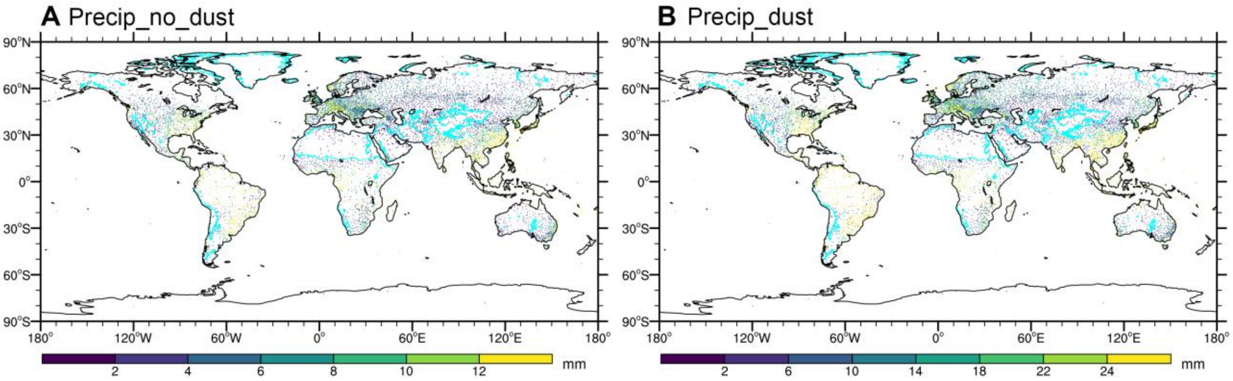

fig. S4. Global spatial distribution of 7-day cumulative precipitation from 1979 to 2023. Panel (**A**) (precip_no_dust) shows precipitation without dust-events, while Panel (**B**) (precip_dust) includes the influence of dust events. The cyan curve outlines the bare soil region.

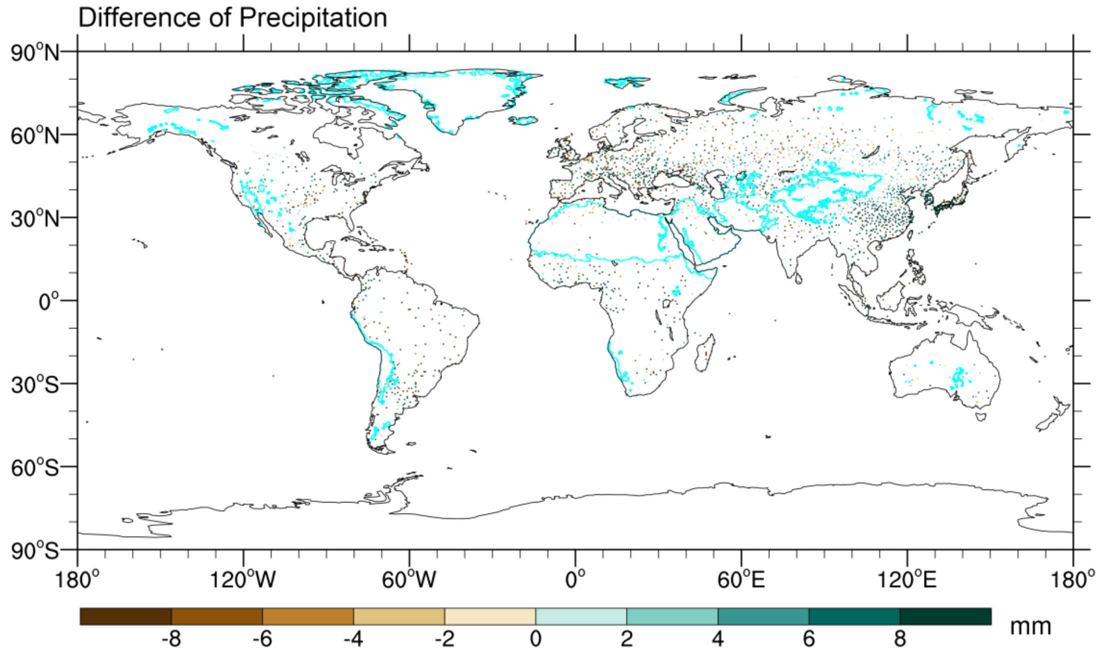

fig. S5. Spatial distribution of 7-day cumulative precipitation differences between dust-active and dust-inactive days. The cyan lines denote the boundaries between dust source regions and transport regions.

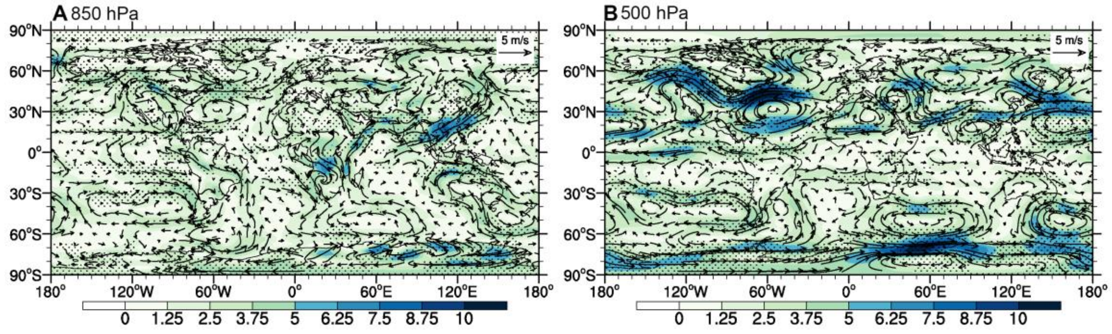

fig. S6. Spatial distributions of anomalous wind fields (m s^{-1}) at (A) 850 hPa and (B) 500 hPa between dust-active and dust-inactive days. Black dots indicate anomalies significant at the 95% confidence level in both pointwise and field significance tests.

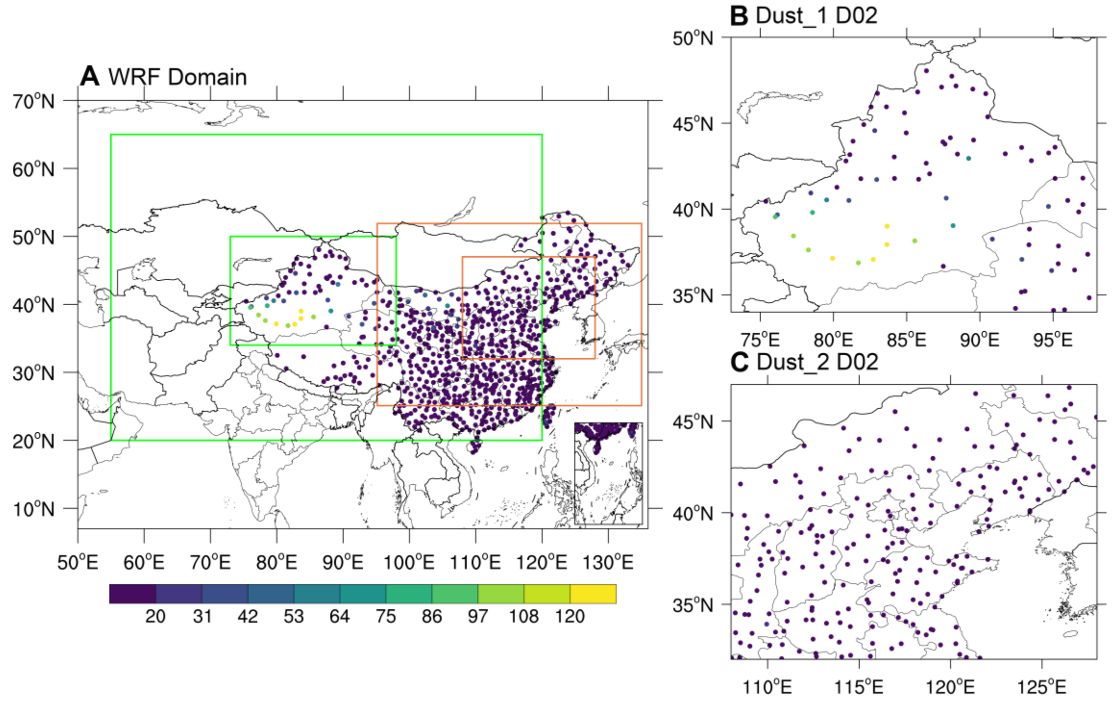

fig. S7. Numerical simulation regions. (**A**) Geographic location of the WRF simulation area in East Asia for Dust_1 (green lines) and Dust_2 (brown lines). (**B**) Domain 02 for Dust_1. (**C**) Domain 02 for Dust_2. Colored dots represent the dust event frequency of site observations.

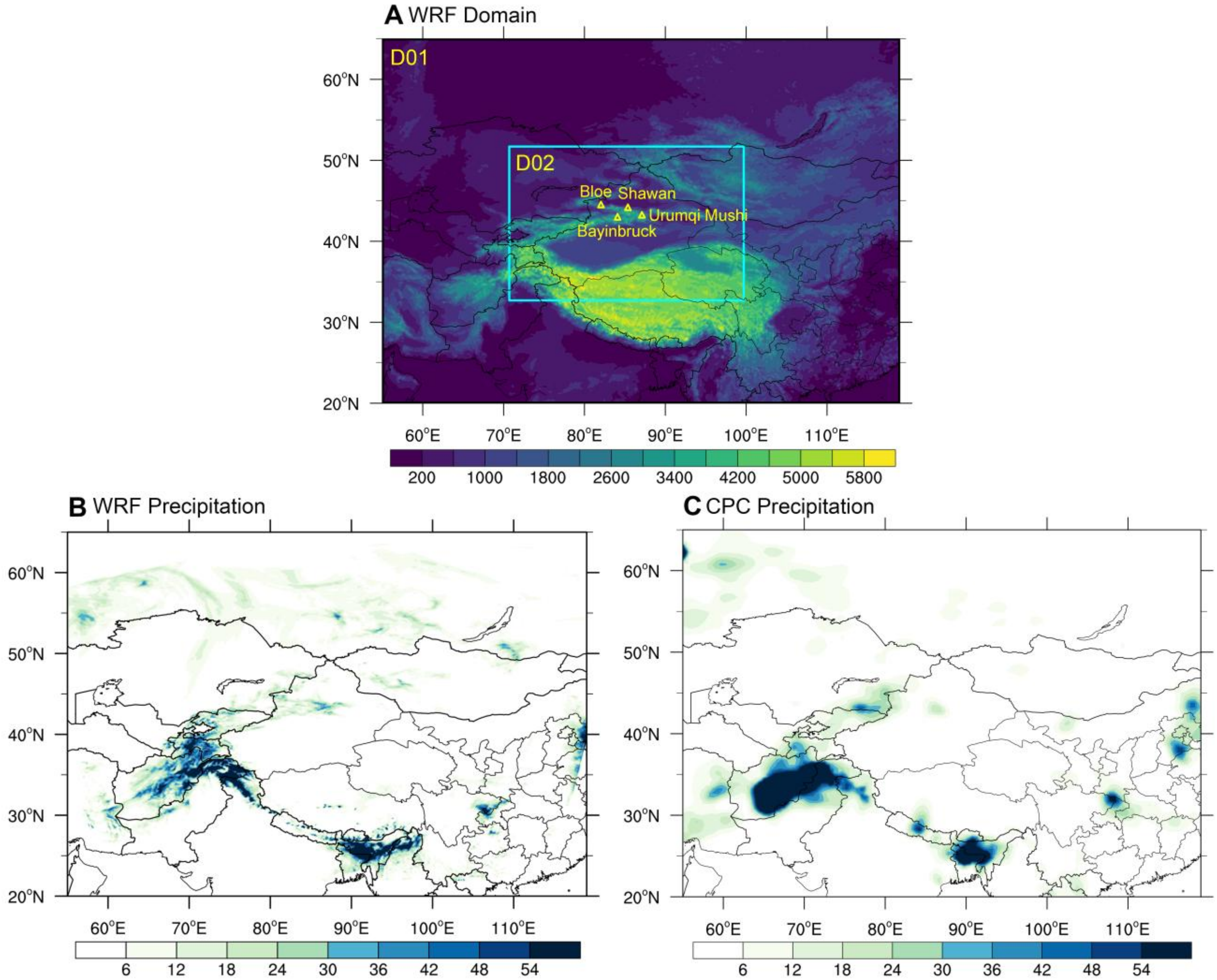

fig. S8. (A) Topographic map of the WRF simulation domain for the period from April 14 to 17, 2020 (Dust_1). (B) Model-simulated and (C) observed spatial distributions of accumulated precipitation (unit: mm). In Panel (**A**), D01 and D02 denote the outer and inner nested domains, respectively, with yellow triangles indicating station locations.

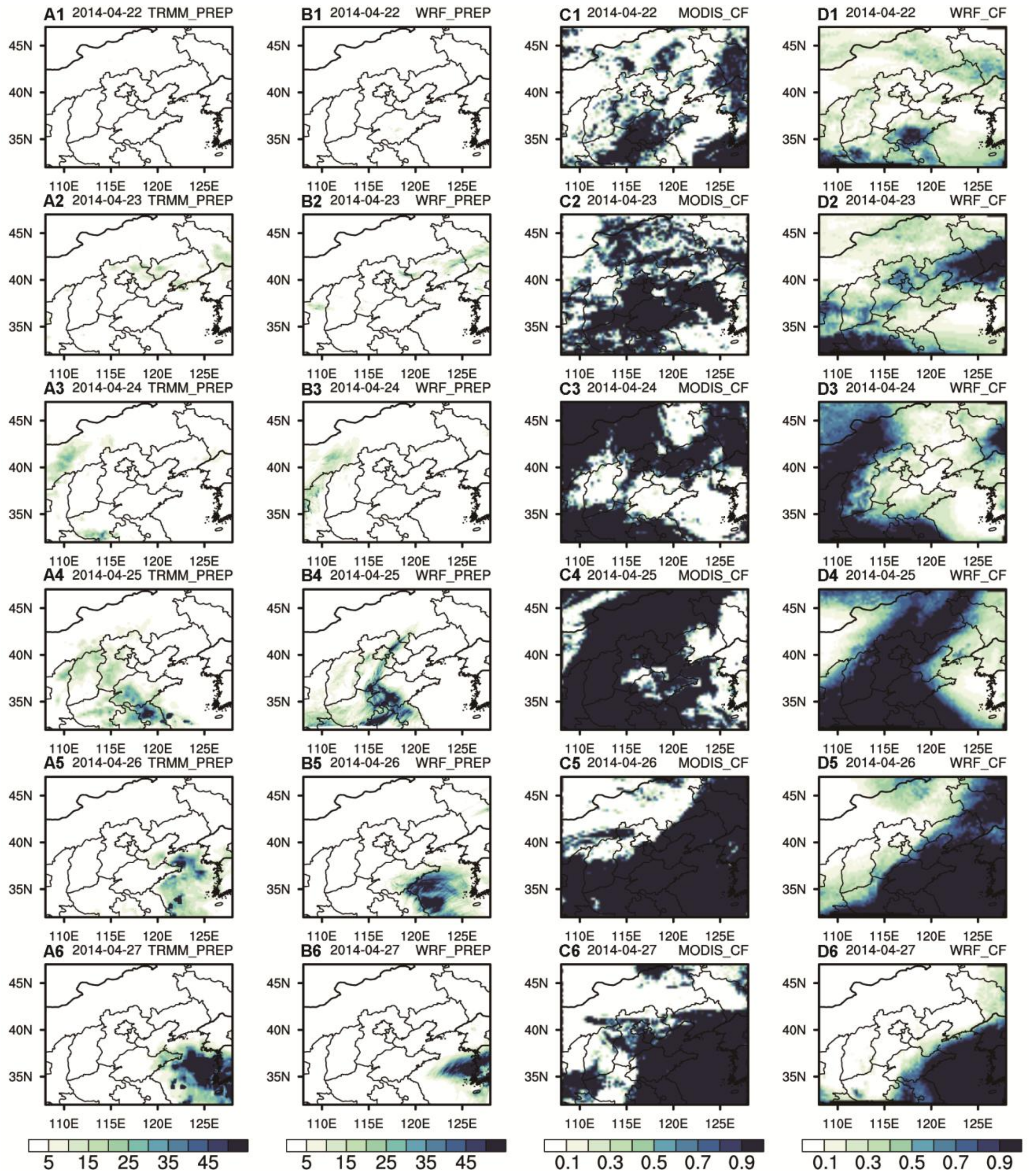

fig. S9. Spatial distributions of (A1–A6) daily accumulated precipitation from TRMM (Tropical Rainfall Measuring Mission satellite) observations, (B1–B6) WRF-simulated daily accumulated precipitation in the D02 domain, (C1–C6) cloud fraction from MODIS, and (D1–D6) WRF-simulated cloud fraction in the D02 domain during April 22–27, 2014 (Dust_2).

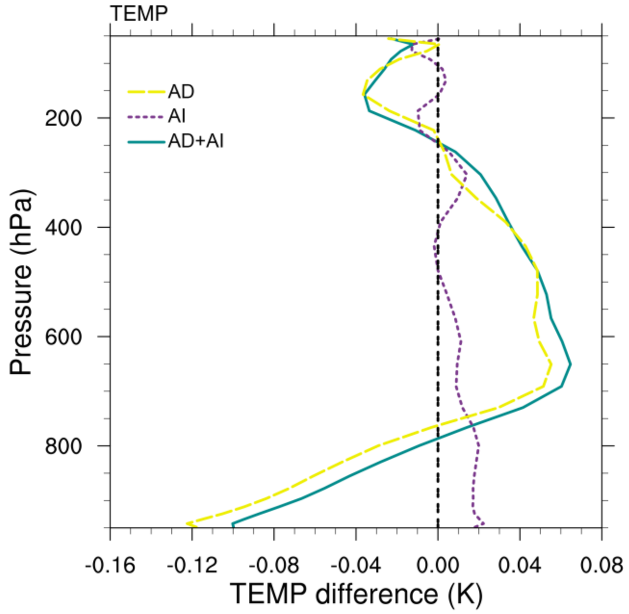

fig. S10. Changes in the regional mean atmospheric temperature profile (units: K) over North China from April 22–27, 2014 (Dust_2), induced by dust aerosols (total effect: cyan line; indirect effect: purple line; direct effect: yellow line).

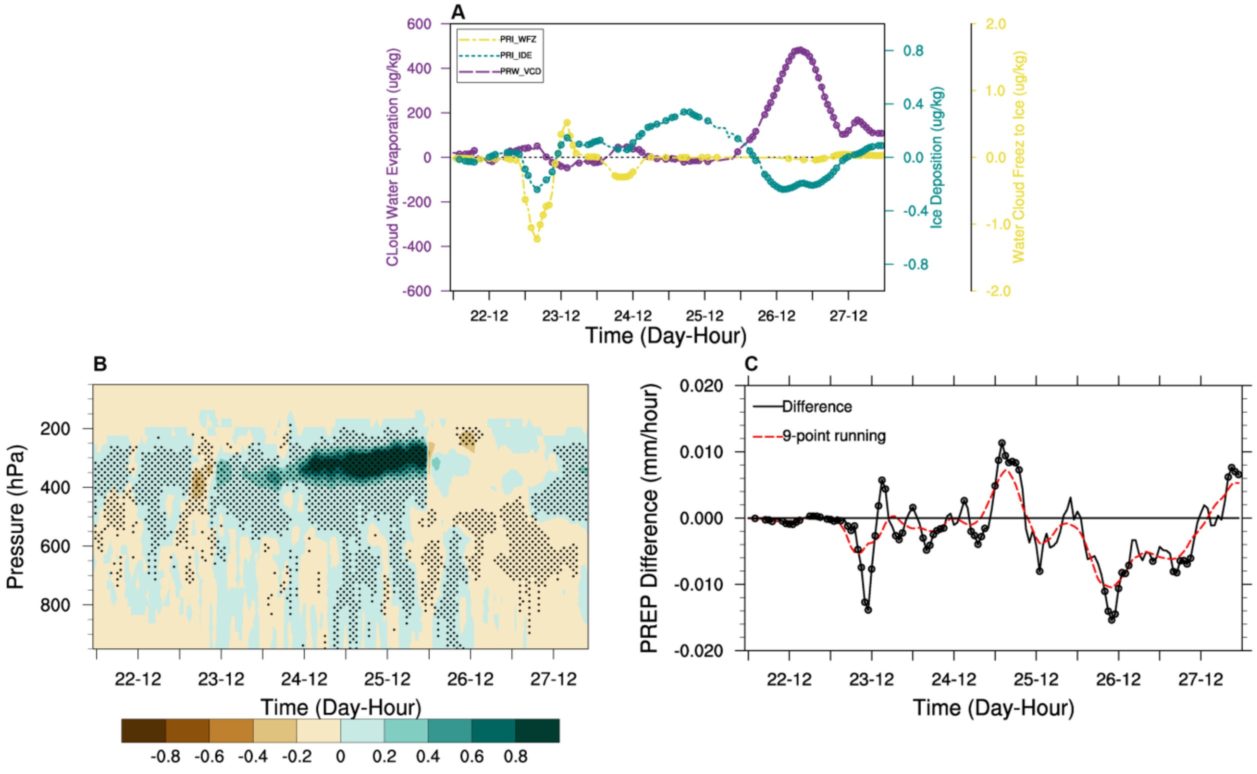

fig. S11. Dust aerosol impacts on cloud microphysics and precipitation from the initial field perturbation experiments. (**A**) Changes in cloud droplet evaporation (purple), ice deposition (cyan), and freezing (yellow) under combined direct and indirect radiative effects. (**B**) Ice crystal number concentration response to combined dust effects. (**C**) Precipitation changes due to dust aerosol direct and indirect effects. In panels A and C, open circles (○) indicate statistical

significance at the 90% confidence level in paired tests, while solid dots (•) in panels B and E denote significance at the 95% confidence level. In panels B, black dots represent results passing paired t-tests at the 95% significance level.

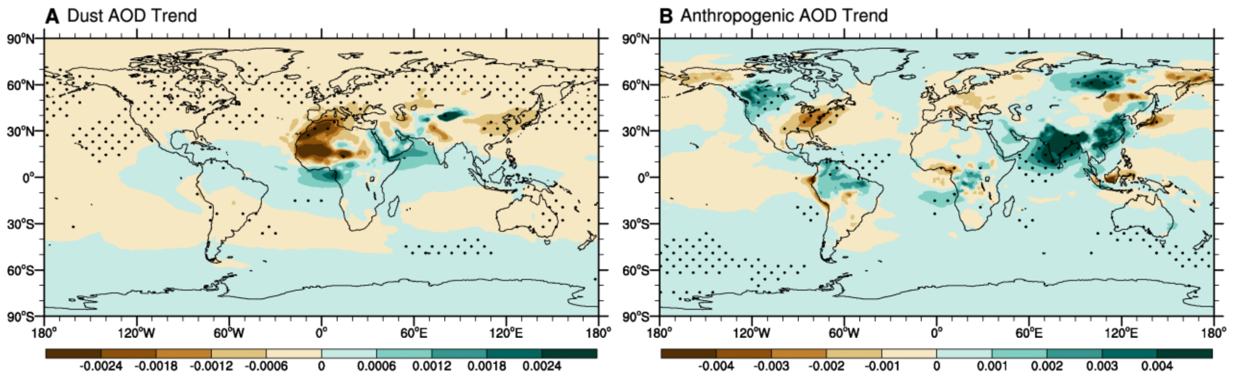

fig. S12. Global AOD trends from 2000 to 2019, showing the spatial distribution of (A) dust aerosol trends and (B) anthropogenic aerosol trends. Black dots mark trends pass the 95% significance test based on the block bootstrap resampling method (the detailed method description is provided in Supplementary text S9).

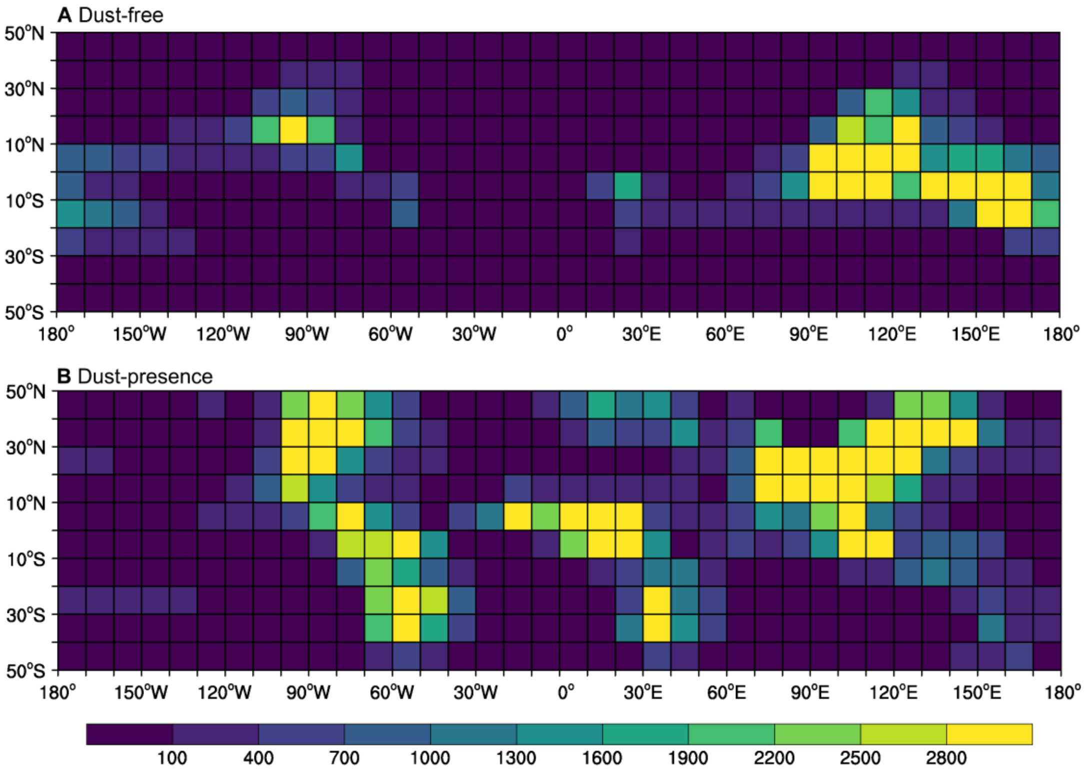

fig. S13. Global spatial patterns of precipitation sampling frequency in (A) dust-free and (B) dust-presence conditions. Grid resolution: 10 °×10 °. The latitudinal range of the sampling is constrained by the coverage of the CPC data.

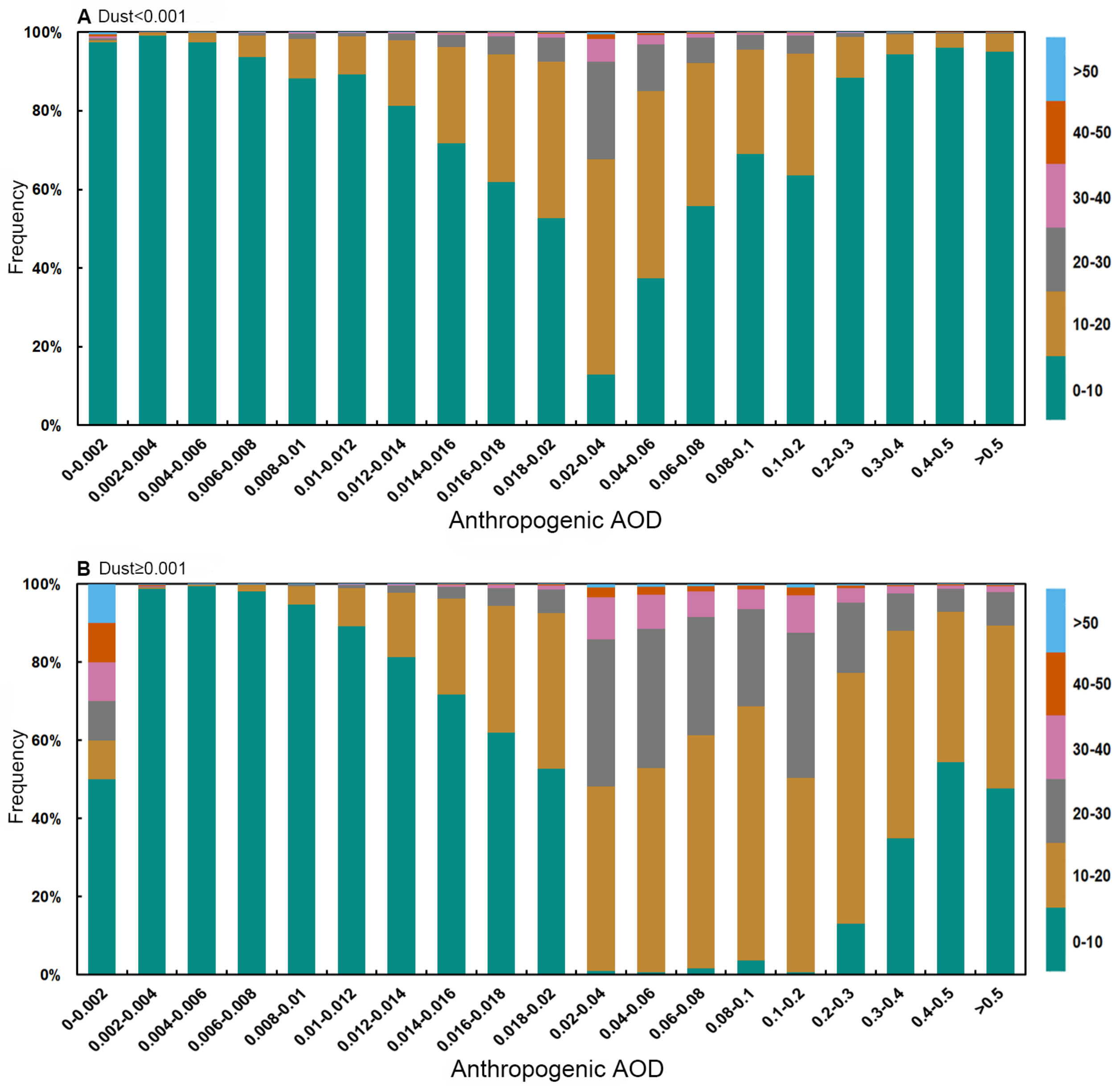

fig. S14. Impacts of dust aerosols on precipitation frequency distributions under varying anthropogenic aerosol loadings. Dust aerosol presence is defined by a threshold AOD of 0.001 (dust-free: AOD < 0.001; dust-presence: AOD ⩾ 0.001).

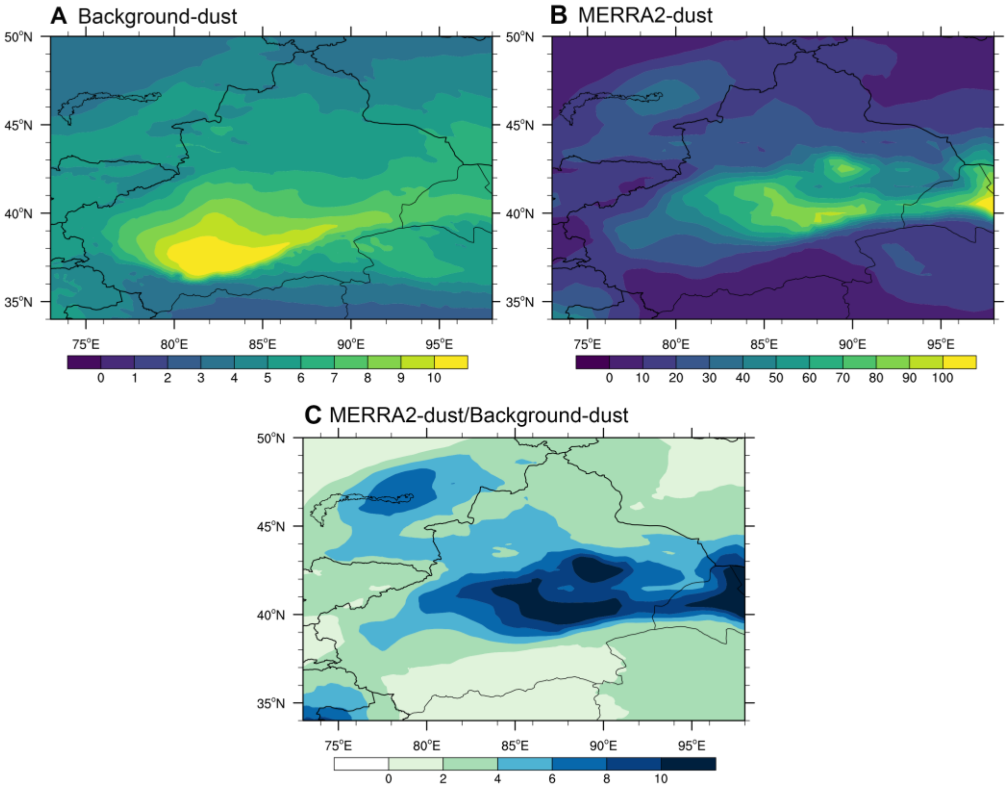

fig. S15. Spatial distributions of the dust number concentrations (×10^{6} kg^{-1}) in (A) control experiment simulation, (B) dust storm case simulation, and (C) the ratio of dust storm case simulation to control experiment simulation.

table S1. Statistics of Global Dust-active and Dust-inactive Days (1979–2023).

**Condition**	**Stations recorded globally in simultaneous observation**					
**Dust-active**	Number of stations	100	150	200	250	300
	Number of days	7,713	2,635	648	161	53
**Dust-inactive**	Number of stations	20,900	20,950	21,000	21,050	21,100
	Number of days	16,104	15,144	11,613	5,247	98

table S2. Model configuration.

Options	Parameter scheme
Microphysics	Thompson aerosol-aware (*69*)
Cumulus convection	Kain-Fritsch (*70,71*)
Longwave and shortwave radiation	RRTMG (Rapid Radiative Transfer Model for General Circulation Models) (*72,73*)
Planetary boundary layer	YSU (Yonsei University) (*74*)
Land surface	5-layer thermal diffusion scheme (*75*)

table S3. Experimental design for dust ensemble simulations.

**Experiment**	**Dust radiative effect**	**Dust concentration**	**Ensemble member size**	**Initial field perturbation details**
Dust_1	Indirect effect	1 × dust (Control)	10	Unbalanced Surface Pressure Perturbation: Variance perturbation coefficient = 0.1<br>Meteorological Field Synchronization: Co-perturbation of temperature/pressure fields<br>Random Seed Recording: put_rand_seed = true<br>Deterministic Perturbation Mode: seed_array1 = 1, seed_array2 = 1
		2 × dust (Sensitivity)	10	Same perturbation methodology as control
Dust_2	Indirect effect	1 × dust (Control)	10	Same perturbation methodology as above
		2 × dust (Sensitivity)	10	Same perturbation methodology as control
	Total effect	1 × dust (Control)	10	Same perturbation methodology as above
		2 × dust (Sensitivity)	10	Same perturbation methodology as control

REFERENCES

1. X. Wang, O. Oenema, W. Hoogmoed, U. Perdok, D. Cai, Dust storm erosion and its impact on soil carbon and nitrogen losses in northern China. *Catena* **66**, 221–227 (2006).

2. D. Griffin, C. Kellogg, Dust storms and their impact on ocean and human health: Dust in Earth's atmosphere. *Ecohealth* **1**, 284–295 (2004).

3. R. Duce, C. Unni, B. Ray, J. Prospero, J. Merrill, Long-range atmospheric transport of soil dust from Asia to the tropical North Pacific: Temporal variability. *Science* **209**, 1522–1524 (1980).

4. T. Fairlie, D. Jacob, R. Park, The impact of transpacific transport of mineral dust in the United States. *Atmos. Environ.* **41**, 1251–1266 (2007).

5. J. Huang, P. Minnis, B. Chen, Z. Huang, Z. Liu, Q. Zhao, Y. Yi, J. K. Ayers, Long-range transport and vertical structure of Asian dust from CALIPSO and surface measurements during PACDEX. *J. Geophys. Res.* **113**, D23212 (2008).

6. A. Slingo, T. Ackerman, R. Allan, E. Kassianov, S. Mcfarlane, G. Robinson, J. Barnard, M. Miller, J. Harries, J. Russell, S. Dewitte, Observations of the impact of a major Saharan dust storm on the atmospheric radiation balance. *Geophys. Res. Lett.* **33**, L24817 (2006).

7. T. Y. Tanaka, Y. Kurosaki, M. Chiba, T. Matsumura, T. Nagai, A. Yamazaki, A. Uchiyama, N. Tsunematsu, K. Kai, Possible transcontinental dust transport from North Africa and the Middle East to East Asia. *Atmos. Environ.* **39**, 3901–3909 (2005).

8. R. Ding, J. Li, S. Wang, F. Ren, Decadal change of the spring dust storm in northwest China and the associated atmospheric circulation. *Geophys. Res. Lett.* **32**, L02808 (2005).

9. T. Shao, Y. Liu, Z. Tan, D. Li, M. Luo, R. Luo, Characteristics and a mechanism of dust weather in northern China. *Climate Dynam.* **61**, 1591–1606 (2022).

10. C. Zhou, Y. Liu, Q. Zhu, Q. He, T. Zhao, F. Yang, W. Huo, X. Yang, A. Mamtimin, In situ observation of warm atmospheric layer and the heat contribution of suspended dust over the Tarim Basin. *Atmos. Chem. Phys.* **22**, 5195–5207 (2022).

11. Z. Liu, A. Omar, M. Vaughan, J. Hair, C. Kittaka, Y. Hu, K. Powell, C. Trepte, D. Winker, C. Hostetler, R. Ferrare, R. Pierce, CALIPSO lidar observations of the optical properties of Saharan dust: A case study of long-range transport. *J. Geophys. Res.* **113**, D07207 (2008).

12. Y. Liu, J. Huang, G. Shi, T. Takamura, P. Khatri, J. Bi, J. Shi, T. Wang, X. Wang, B. Zhang, Aerosol optical properties and radiative effect determined from sky-radiometer over Loess Plateau of Northwest China. *Atmos. Chem. Phys.* **11**, 11455–11463 (2011).

13. Y. Liu, R. Jia, T. Dai, Y. Xie, G. Shi, A review of aerosol optical properties and radiative effects. *J. Meteor. Res.* **28**, 1003–1028 (2014).

14. J. Huang, Q. Fu, J. Su, Q. Tang, P. Minnis, Y. Hu, Y. Yi, Q. Zhao, Taklimakan dust aerosol radiative heating derived from CALIPSO observations using the Fu-Liou radiation model with CERES constraints. *Atmos. Chem. Phys.* **9**, 4011–4021 (2009).

15. J. Li, B. Carlson, Y. Yung, D. Lv, J. Hansen, J. Penner, H. Liao, V. Ramaswamy, R. Kahn, P. Zhang, O. Dubovik, A. Ding, A. Lacis, L. Zhang, Y. Dong, Scattering and absorbing aerosols in the climate system. *Nat. Rev. Earth Environ.* **3**, 363–379 (2022).

16. Y. Sun, C. Zhao, Distinct impacts on precipitation by aerosol radiative effect over three different megacity regions of eastern China. *Atmos. Chem. Phys.* **21**, 16555–16574 (2021).

17. C. Zhao, Y. Yang, H. Fan, J. Huang, Y. Fu, X. Zhang, S. Kang, Z. Cong, H. Letu, M. Menenti, Aerosol characteristics and impacts on weather and climate over the Tibetan Plateau. *Natl. Sci. Rev.* **7**, 492–495 (2020).

18. A. Li, C. Shi, S. Yin, N. Li, H. Letu, G. Shi. Variation of surface solar radiation components from 2016 to 2020 in China: Perspective from geostationary satellite observation with a high spatiotemporal resolution. *Sci. Total Environ.* **954**, 176264 (2024).

19. J. Penner, D. Hegg, R. Leaitch, Unraveling the role of aerosols in climate change. *Environ. Sci. Technol.* **35**, 332–340 (2001).

20. S. Twomey, The influence of pollution on the shortwave albedo of clouds. *J. Atmos. Sci.* **34**, 1149–1152 (1977).

21. D. Rosenfeld, S. Sherwood, R. Wood, L. Donner, Climate effects of aerosol-cloud interactions. *Science* **343**, 379–380 (2014).

22. C. Zhao, Y. Lin, F. Wu, Y. Wang, Z. Li, D. Rosenfeld, Y. Wang, Enlarging rainfall area of tropical cyclones by atmospheric aerosols. *Geophys. Res. Lett.* **45**, 8604–8611 (2018).

23. C. Zhao, Y. Qiu, X. Dong, Z. Wang, Y. Peng, B. Li, Z. Wu, Y. Wang, Negative aerosol-cloud r_e relationship from aircraft observations over Hebei, China. *Earth Space Sci.* **5**, 19–29 (2018).

24. C. Zhao, T. J. Garrett, Effects of Arctic haze on surface cloud radiative forcing. *Geophys. Res. Lett.* **42**, 557–564 (2015).

25. Y. Liu, Q. Zhu, J. Huang, S. Hua, R. Jia, Impact of dust-polluted convective clouds over the Tibetan Plateau on downstream precipitation. *Atmos. Environ.* **209**, 67–77 (2019).

26. Y. Liu, S. Hua, R. Jia, J. Huang, Effect of aerosols on the ice cloud properties over the Tibetan Plateau. *J. Geophys. Res. Atmos.* **124**, 9594–9608 (2019).

27. Y. Liu, Q. Zhu, S. Hua, A. Khan, T. Dai, Y. Cheng, Tibetan Plateau driven impact of Taklimakan dust on northern rainfall. *Atmos. Environ.* **234**,117583 (2020).

28. Y. Liu, Y. Li, J. Huang, Q. Zhu, S. Wang, Attribution of the Tibetan Plateau to northern drought. *Natl. Sci. Rev.* **7**, 489–492 (2020).

29. Y. Liu, J. Huang, T. Wang, J. Li, H. Yan, Y. He, Aerosol-cloud interactions over the Tibetan Plateau: An overview. *Earth Sci. Rev.* **234**, 104216 (2022).

30. Y. Liu, T. Shao, S. Hua, Q. Zhu, R. Luo, Association of anthropogenic aerosols with subtropical drought in East Asia. *Int. J. Climatol.* **40**, 3500–3513 (2020).

31. R. Jia, Y. Liu, S. Hua, Q. Zhu, T. Shao. Estimation of the aerosol radiative effect over the Tibetan Plateau based on the latest CALIPSO product. *J. Meteorol. Res.* **32**, 707–722 (2018).

32. A. McComiskey, G. Feingold, The scale problem in quantifying aerosol indirect effects. *Atmos. Chem. Phys.* **12**, 1031–1049 (2012).

33. J. Fan, Y. Wang, D. Rosenfeld, X. Liu, Review of aerosol-cloud interactions: Mechanisms, significance and challenges. *J. Atmos. Sci.* **73**, 4221–4252 (2016).

34. F. Glassmeier, F. Hoffmann, J. Johnson, T. Yamaguchi, K. Carslaw, G. Feingold, Aerosol-cloud-climate cooling overestimated by ship-track data. *Science* **371**, 485 – 489 (2021).

35. A. Arola, A. Lipponen, P. Kolmonen, T. H. Virtanen, N. Bellouin, D. P. Grosvenor, E. Gryspeerdt, J. Quaas, H. Kokkola, Aerosol effects on clouds are concealed by natural cloud heterogeneity and satellite retrieval errors. *Nat. Commun.* **13**, 7357 (2022).

36. J. Huang, P. Minnis, H. Yan, Y. Yi, B. Chen, L. Zhang, J. K. Ayers, Dust aerosol effect on semi-arid climate over Northwest China detected from A-Train satellite measurements. *Atmos. Chem. Phys.* **10**, 6863–6872 (2010).

37. R. Luo, Y. Liu, M. Luo, D. Li, Z. Tan, T. Shao, A. Khan, Dust effects on mixed-phase clouds and precipitation during a super dust storm over northern China. *Atmos. Environ.* **313**, 120081 (2023).

38. R. Sequeira, On the large-scale impact of arid dust on precipitation chemistry of the continental northern hemisphere. *Atmos. Environ. Part A Gen. Top.* **27**, 1553 – 1565 (1993).

39. T. Shao, Y. Liu, R. Wang, Q. Zhu, Z. Tan, R. Luo, Role of anthropogenic aerosols in affecting different-grade precipitation over eastern China: A case study. *Sci. Total Environ.* **807**, 150886 (2022).

40. U. Dusek, M. Frank, P. Hildebrandt, L. Curtius, J. Schneider, S. Walter, D. Chand, F. Drewnick, S. Hings, D. Jung, S. Borrmann, M. O. Andreae, Size matters more than chemistry for cloud-nucleating ability of aerosol particles. *Science* **312**, 1375–1378 (2006).

41. H. Xue, G. Feingold, B. Stevens, Aerosol effects on clouds, precipitation, and the organization of shallow cumulus convection. *J. Atmos. Sci.* **65**, 392–406 (2008).

42. E. Erfani, P. Blossey, R. Wood, J. Mohrmann, S. J. Doherty, M. Wyant, K.-T. O. Simulating aerosol lifecycle impacts on the subtropical stratocumulus-to-cumulus transition using large-eddy simulations. *J. Geophys. Res. Atmos.* **127**, e2022JD037258 (2022).

43. S. M. Calderón, J. Tonttila, A. Buchholz, J. Joutsensaari, M. Komppula, A. Leskinen, L. Hao, D. Moisseev, I. Pullinen, P. Tiitta, J. Xu, A. Virtanen, H. Kokkola, S. Romakkaniemi. Aerosol–stratocumulus interactions: Towards a better process understanding using closures between observations and large eddy simulations. *Atmos. Chem. Phys.* **22**, 12417–12441 (2022).

44. A. Khain, D. Rosenfeld, A. A. Pokrovsky. Aerosol impact on the dynamics and microphysics of deep convective clouds. *Q. J. Roy. Meteorol. Soc.* **131**, 2639–2663 (2005).

45. J. Fan, D. Rosenfeld, Y. Zhang, S. E. Giangrande, Z. Li, L. A. T. Machado, S. T. Martin, Y. Yang, J. Wang, P. Artaxo, H. M. J. Barbosa, R. Braga, J. M. Comstock, Z. Feng, W. Gao, H. B. Gomes, F. Mei, C. Pöhlker, M. L. Pöhlker, U. Pöschl, R. A. F. D. Souza. Substantial convection and precipitation enhancements by ultrafine aerosol particles. *Science* **359**, 411–418 (2018).

46. Y. Qian, D. Gong, J. Fan, L. R. Leung, R. Bennartz, D. Chen, W. Wang. Heavy pollution suppresses light rain in China: Observations and modeling. *J. Geophys. Res.* **114**, D00K02 (2009).

47. X. Liu, X. Xie, Z. Yin, C. Liu, A. Gettelman. A modeling study of the effects of aerosols on clouds and precipitation over East Asia. *Theor. Appl. Climatol.* **106**, 343–354 (2011).

48. J. Guo, M. Deng, S. S. Lee, F. Wang, Z. Li, P. Zhai, H. Liu, W. Lv, W. Yao, X. Li. Delaying precipitation and lightning by air pollution over the Pearl River Delta. Part I: Observational analyses. *J. Geophys. Res. Atmos.* **121**, 6472–6488 (2016).

49. J. Fan, R. Zhang, W.-K. Tao, K. I. Mohr. Effects of aerosol optical properties on deep convective clouds and radiative forcing. *J. Geophys. Res.* **113**, D08209 (2008).

50. D. Rosenfeld, U. Lohmann, G. B. Raga, C. D. O'Dowd, M. Kulmala, S. Fuzzi, A. Reissell, M. O. Andreae. Flood or drought: How do aerosols affect precipitation? *Science* **321**, 1309–1313 (2008).

51. I. Koren, Y. J. Kaufman, L. A. Remer, J. V. Martins. Measurement of the effect of Amazon smoke on inhibition of cloud formation. *Science* **303**, 1342–1345 (2004).

52. S. Menon, J. Hansen, L. Nazarenko, Y. Luo. Climate effects of black carbon aerosols in China and India. *Science* **297**, 2250–2253 (2002).

53. F. Giorgi, X. Bi, Y. Qian, Direct radiative forcing and regional climatic effects of anthropogenic aerosols over East Asia: A regional coupled climate-chemistry/aerosol model study. *J. Geophys. Res.* **107**, 4439 (2002).

54. Q. Min, R. Li, B. Lin, E. Joseph, S. Wang, Y. Hu, V. Morris, F. Chang, Evidence of mineral dust altering cloud microphysics and precipitation. *Atmos. Chem. Phys.* **19**, 3223–3231 (2009).

55. I. Koren, Y. J. Kaufman, D. Rosenfeld, L. A. Remer, Y. Rudich, Aerosol invigoration and restructuring of Atlantic convective clouds. *Geophys. Res. Lett.* **32**, L14808 (2005).

56. K. M. Lau, M. K. Kim, K. M. Kim, Asian summer monsoon anomalies induced by aerosol direct forcing: The role of the Tibetan Plateau. *Clim. Dyn.* **26**, 855–864 (2006).

57. V. Vinoj, P. J. Rasch, H. Wang, J. Yoon, P. Ma, K. Landu, B. Singh, Short-term modulation of Indian summer monsoon rainfall by West Asian dust. *Nat. Geosci.* **7**, 308–313 (2014).

58. F. Solmon, V. S. Nair, M. Mallet, Increasing Arabian dust activity and the Indian summer monsoon. *Atmos. Chem. Phys.* **15**, 8051–8064 (2015).

59. P. Knippertz, M. C. Todd, Mineral dust aerosols over the Sahara: Meteorological controls on emission and transport and implications for modeling. *Rev. Geophys.* **50**, RG1007 (2012).

60. A. Prein, P. Mooney, J. Done. The multi-scale interactions of atmospheric phenomenon in mean and extreme precipitation. *Earth's Future* **11**, e2023EF003534 (2023).

61. B. Dieppois, A. Diedhiou, A. Durand, M. Fournier, N. Massei, D. Sebag, Y. Xue, B. Fontaine, Quasi-decadal signals of Sahel rainfall and West African monsoon since the mid-twentieth century. *J. Geophys. Res. Atmos.* **118**, 12587–12599 (2013).

62. R. E. Livezey, W. Y. Chen, Statistical field significance and its determination by Monte Carlo technique. *Mon. Weather Rev.* **111**, 46–59 (1983).

63. D. S. Wilks, "The stippling shows statistically significant grid points": How research results are routinely overstated and overinterpreted, and what to do about it. *Bull. Am. Meteorol. Soc.* **97**, 2263–2273 (2016).

64. D. R. Stratman, N. Yussouf, C. A. Kerr, B. C. Matilla, J. R. Lawson, Y. Wang, Testing stochastic and perturbed parameter methods in an experimental 1-km warn-on-forecast system using NSSL's phased-array radar observations. *Mon. Weather Rev.* **152**, 433–454 (2024).

65. S. Wang, J. Min, X. Li, X. Qiao, An atmospheric instability perturbation approach for ensemble forecasts and its application in heavy rain cases. *J. Adv. Model. Earth Syst.* **17**, e2024MS004556 (2025).

66. M. Mudelsee, Statistical analysis in climate research. *Comput. Geosci.* **27**, 371–373 (2001).

67. S. N. Lahiri, Theoretical comparisons of block bootstrap methods. *Ann. Stat.* **27**, 386–404 (1999).

68. P. Hall, J. L. Horowitz, B.-Y. Jing, On blocking rules for the bootstrap with dependent data. *Biometrika* **82**, 561–574 (1995).

69. G. Thompson, T. Eidhammer, A study of aerosol impacts on clouds and precipitation development in a large winter cyclone. *J. Atmos. Sci.* **71**, 3636–3658 (2014).

70. J. Kain, J. Fritsch, A one-dimensional entraining/detraining plume model and its application in convective parameterization. *J. Atmos. Sci.* **47**, 2784–2802 (1990).

71. J. Kain, The Kain-Fritsch convective parameterization: An update. *J. Appl. Meteorol.* **43**, 170–181 (2004).

72. E. Mlawer, S. Taubman, P. Brown, M. J. Iacono, S. A. Clough, Radiative transfer for inhomogeneous atmospheres: RRTM, a validated correlated-k model for the longwave. *J. Geophys. Res.* **102**, 16663–16682 (1997).

73. M. Iacono, E. Mlawer, S. Clough, J. Morcrette, Impact of an improved longwave radiation model, RRTM, on the energy budget and thermodynamic properties of the NCAR community climate model, CCM3. *J. Geophys. Res.* **105**, 14873–14890 (2000).

74. S. Hong, Y. Noh, J. Dudhia, A new vertical diffusion package with an explicit treatment of entrainment processes. *Mon. Weather Rev.* **134**, 2318–2341 (2006).

75. J. Dudhia, A multi-layer soil temperature model for MM5. *Proc. 6th PSU/NCAR Mesoscale Model Users' Workshop* (1996).
